# The redox rhythm gates immune-induced cell death distinctly from the genetic clock

**DOI:** 10.1073/pnas.2519251122

**Published:** 2025-09-10

**Authors:** Sargis Karapetyan, Musoki Mwimba, Tianyuan Chen, Zhujun Yao, Xinnian Dong

**Affiliations:** ^a^Department of Biology, Duke University, Durham, NC 27708; ^b^HHMI, Duke University, Durham, NC 27708

**Keywords:** plant immunity, circadian clock, redox rhythm, programmed cell death, metabolism

## Abstract

Organisms use circadian clocks to synchronize physiological processes to anticipate the Earth’s day-night cycles and regulate responses to environmental signals to gain competitive advantage. While divergent genetic clocks have been studied extensively in bacteria, fungi, plants, and animals, an ancient conserved circadian redox rhythm has been recently reported. However, its biological function and physiological outputs remain elusive. Here, we uncovered the coexistence of redox and genetic rhythms with distinct period lengths and transcriptional targets through concurrent metabolic and transcriptional time-course measurements in an *Arabidopsis* long-period clock mutant. Analysis of the target genes indicated regulation of the immune-induced programmed cell death (PCD) by the redox rhythm. Moreover, this time-of-day-sensitive PCD was eliminated by redox perturbations and by blocking the signaling pathway of the plant defense hormones jasmonic acid/ethylene, while remaining intact in genetic clock-defective backgrounds. This study shows that compared to robust genetic clocks, the more sensitive circadian redox rhythm serves as a signaling hub in regulating incidental energy-intensive processes, such as immune-induced PCD involving reprogramming of chloroplast and mitochondria activities, to provide organisms a flexible strategy to mitigate metabolic overload during stress responses.

## Significance

The redox rhythm is a conserved nontranscriptional circadian oscillation first discovered in anucleate red blood cells. However, the lack of a distinct physiological response regulated by the redox rhythm impeded the understanding of its biological function. By using an *Arabidopsis* long-period genetic clock mutant, we distinguished the redox rhythm and the genetic clock based on their difference in period length and identified immune-induced programmed cell death (PCD) as a physiological output of the redox rhythm. By using genetic and chemical perturbations, we further demonstrated the biological role of the redox rhythm in controlling incidental energy-intensive physiological responses, such as PCD, distinctly from the genetic clock.

Rhythmic daily behaviors in plants and animals have been observed since antiquity (1). However, the mechanistic basis of circadian clocks was not revealed until the 20th century, with the discoveries of first clock genes (2–4). These genes encode an internal genetic oscillator consisting of transcription–translation feedback loops (TTFL), which synchronizes the physiological processes/behaviors with diurnal environmental changes and “gates” (i.e., modulates the strength of) responses to biotic and abiotic changes to improve fitness of the organism (5, 6). In addition to the genetic clock, discovery of circadian oscillations of peroxiredoxins (PRX) in their over- and hyperoxidized forms (PRX-SO_2/3_) along with a number of periodic transcripts in backgrounds lacking oscillations of the canonical TTFL circuitry (7–9) suggest the existence of a parallel nontranscriptional oscillator. Furthermore, the conservation of this redox rhythm across all lineages of life indicates its possible importance throughout evolution (9). Additionally, while the redox rhythm was initially determined by the oscillations of PRX-SO_2/3_, it became increasingly evident that the redox rhythm extends well beyond PRX and is better defined as the oscillation of the overall cellular redox metabolism (10) including key electron carriers NAD(P)H (11, 12) and glutathione (10). However, despite the multiple attempts to characterize the oscillator driving the redox rhythm (13–16), the results were inconclusive. Moreover, after the initial excitement of these ground-breaking discoveries, concerns over the observed oscillations in genetic clock-defective mutants being driven by unknown external factors (17), along with the lack of a clear physiological output regulated specifically by the circadian redox rhythm, have raised questions about the autonomy and the significance of the redox oscillation. Therefore, although an interplay between the genetic clock and the redox rhythm has been well established (12, 14, 15), the biological function of the circadian redox oscillation remains largely unknown.

## Results

### The Genetic Clock and Redox Rhythm Are Disentangled in a Long-Period Clock Mutant.

The difficulty in elucidating the distinct circadian function of the redox rhythm stems from the fact that, in addition to the lack of the knowledge of its oscillatory mechanism, it is intricately intertwined with the genetic clock. Disruption of the redox rhythm can affect the period, the phase, or the amplitude of the genetic clocks depending on their molecular architectures and the nature of the disruption (12, 14, 15) and vice versa (9). Thus far, genetic clock-defective mutants have been used in an attempt to identify the specific transcriptional targets of the redox rhythm. However, the scant overlap between the oscillating transcripts in these mutants and the wild type (WT) (7, 8) raised the concern that the targets identified in the mutants might not be functionally relevant for the WT (17).

To test whether the genetic and redox rhythms can be disentangled by an alternative method, we used the *Arabidopsis* mutant of the clock genes *PSEUDO-RESPONSE REGULATORs* (*PRRs*) *7* and *9* (*prr7 prr9*) which has the intrinsic genetic clock periods of approximately 24 h at 12 °C, 32 h at 22 °C (typical temperature for growing *Arabidopsis*), and 36 h at 30 °C (18). Therefore, in contrast to the WT, which has the ability to maintain the ~24 h period across a physiological range of temperatures, the *prr7 prr9* mutant is defective in temperature compensation. This temperature-dependent period variation in the *prr7 prr9* mutant makes it a tractable system to separate the redox and genetic rhythms based on oscillation periods.

**Fig. 1.**
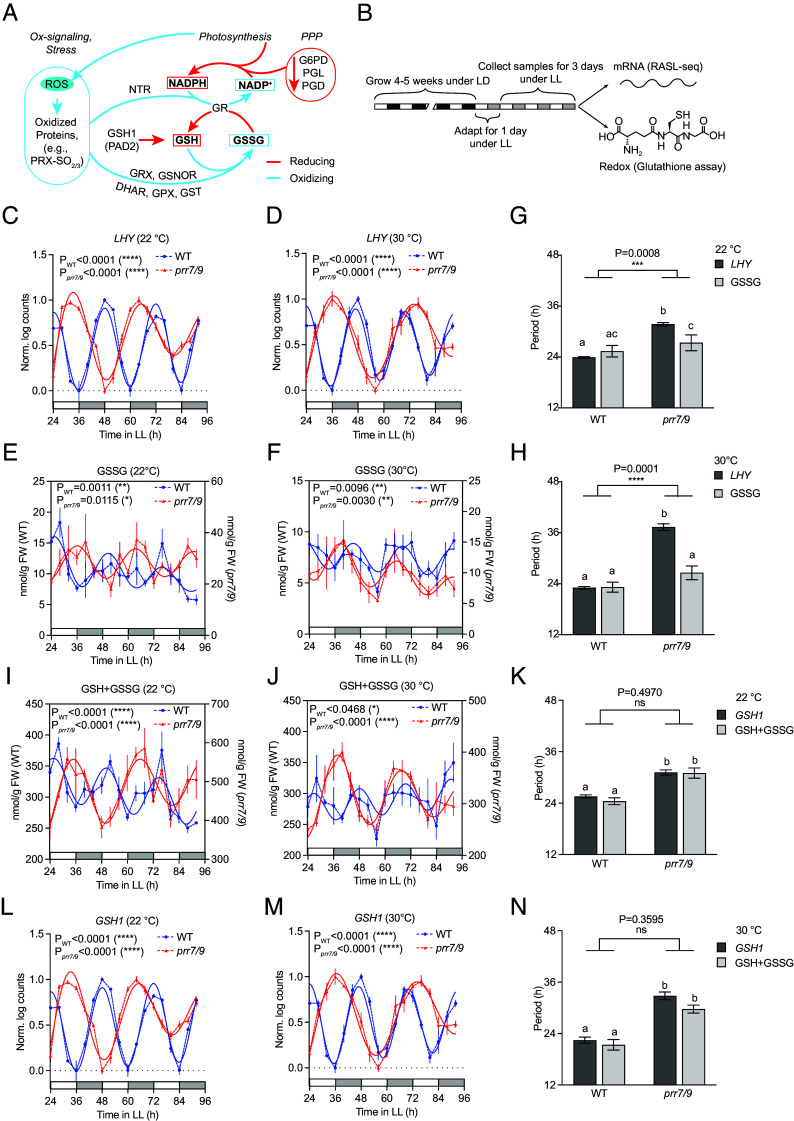
The redox rhythm and genetic clock are disentangled in the *prr7 prr9* double mutant. (*A*) The role of glutathione in plant redox metabolism. In plants, photosynthesis and oxidative signaling/stress lead to oxidation of proteins, including the redox rhythm markers, PRX-SO_2/3_, by ROS or reactive nitrogen species. The oxidized proteins can be reduced through either the thioredoxin/NTR system using NADPH, or GRX with GSH and through S-nitrosoglutathione reductase (GSNOR) also with GSH. GSH is directly oxidized during ROS detoxification either by glutathione peroxidase or dehydroascorbate reductase as a part of ascorbate-glutathione cycle. GSH is also consumed through the activity of glutathione S-transferases. The reducing power used in these reactions is generated during photosynthesis or through oxidative PPP catalyzed by G6PD, phosphogluconolactonase (PGL), and phosphogluconate dehydrogenase. GSH production is rate-limited by the single enzyme, GLUTATHIONE SYNTHSE1 (GSH1) also known as PAD2 and is regenerated by GR using NADPH. Red arrows represent reactions that shift the equilibrium toward GSH/NADPH from GSSG/NADP^+^ (“reducing”), with cyan arrows representing the opposite (“oxidizing”). (*B*) Workflow of the sample collection and processing for transcriptional and metabolic measurements. WT and *prr7 prr9* (*prr7/9*) plants were first grown under LD-entraining conditions for 4 to 5 wk and then released to constant light (LL) conditions. After 24 h of adaptation, samples were collected concurrently for mRNA (RASL-seq) and redox (GSH/GSSG) measurements. (*C* and *D*) Normalized *LHY* transcript levels in WT and *prr7/9* at 22 °C (*C*) and 30 °C (*D*). (*E* and *F*) Oxidized glutathione (GSSG) levels in WT and *prr7/9* at 22 °C (*E*) and 30 °C (*F*). Solid lines represent the harmonic regression, indicating statistically significant oscillations. (*G* and *H*) Estimated periods from the harmonic regression for (*C–F*). Individual values were compared using Tukey’s multiple comparison test, while the interaction was tested using two-way ANOVA; letters indicate statistically significant differences, ns, not significant. (*I-N*) Total glutathione (GSH+GSSG) and normalized *GSH1* transcript levels in WT and *prr7/9* at 22 °C (*I*, *K,* and *L*) and 30 °C (*J*, *M,* and *N*) analyzed as in (*C–H*). n = 3 for (*D*, *E*, *I,* and *M*), n = 4 or 5 for (*F* and *J*) and n = 5 or 6 biological replicates per timepoint for (*C* and *L*). All values are means ± SEM.

We used oxidized (GSSG) glutathione to measure the period of the redox rhythm for several reasons. First, GSSG provides a good approximation of the sum total of glutathione redox metabolism (i.e., a branch of the redox rhythm), because unlike GSH, which can be synthesized de novo, GSSG is produced solely during cellular redox reactions. Unsurprisingly, GSSG was previously shown to follow the same redox rhythm as peroxiredoxin oscillations which were detected in all lineages of life (9, 10). Second, relatively high glutathione abundance allows more robust measurements. Finally, glutathione plays more diverse roles in maintaining cellular redox homeostasis in plants (19) compared to other electron carriers, such as NADPH. While NADPH can be directly utilized for protein reduction by the thioredoxin-NADPH-dependent thioredoxin reductase (NTR) system, a significant portion of it is used to reduce GSSG to GSH via glutathione reductase (GR) to mitigate oxidative stress. GSH is not only used to reduce oxidized proteins but also plays a central role in direct scavenging of reactive oxygen species (ROS) (Fig. 1*A*). A more detailed overview of the redox regulation in plants can be found in the recent reviews (20, 21).

Prior to sample collection, we grew WT and *prr7 prr9* plants in temperature- and humidity-controlled Percival chambers (22 °C, 65% RH) (22), under 12 h light/12 h dark (LD) and then released the plants to constant light (LL) conditions at either 22 °C or 30 °C to collect plant tissue for concurrent glutathione and transcriptomic analyses from 24 h to 92 h in LL (Fig. 1*B*). For transcriptomic measurements, we used the multiplex RNA annealing selection ligation-sequencing (RASL-seq) approach (23) to analyze the expression of a selected pool of ~700 genes (Dataset S1) as an alternative to RNA-seq to accommodate the large sample number for the time-course experiments (two genotypes, two temperatures, and 18 time points with 6 replicates for 22 °C and 3 replicates for 30 °C).

Harmonic regression analysis of the time-course data (12) revealed that in contrast to the core genetic clock gene *LATE ELONGATED HYPOCOTYL (LHY)* which showed longer periods in the *prr7 prr9* mutant than in the WT (Fig. 1 *C* and *D*), GSSG exhibited close-to-WT periods in the *prr7 prr9* mutant, albeit with different phases, at both 22 °C and 30 °C (Fig. 1 *E* and *F*). Thus, the redox rhythm, represented by GSSG, is distinguished from the genetic clock based on the period lengths in the *prr7 prr9* mutant (Fig. 1 *G* and *H*). Notably, while the elevated temperature increased the period of *LHY* from 31.71 h to 37.34 h in the *prr7 prr9* mutant, recapitulating the previously observed temperature overcompensation (18), the period of GSSG changed only slightly from 27.34 h to 26.57 h. Therefore, GSSG oscillation appears to exhibit temperature compensation, a known hallmark of circadian clocks (24), separately from the genetic clock.

In contrast to GSSG, the total glutathione (GSSG + GSH) oscillated with the genetic clock, likely through the regulation of the *GLUTAMATE-CYSTEINE LIGASE* (*GSH1*), also known as *PHYTOALEXIN DEFICIENT 2* (*PAD2*) (25) (Fig. 1 *A* and *I*–*N*). This gene encodes the enzyme that catalyzes the rate-limiting step of glutathione biosynthesis in *Arabidopsis* (26) with its rhythmic pattern aligned with that of total glutathione. Since the total glutathione and GSSG levels were measured in the same samples, the separation of GSH+GSSG and GSSG oscillations further supports the independence of the redox rhythm from the genetic clock. We hypothesize that since GSSG is produced only through redox metabolism and is the much smaller and more dynamic fraction of the total glutathione pool (~2 to 5%; Fig. 1 *E*, *F*, *I*, and *J*) (27, 28), it may serve as an “indicator” of GSH oxidation reactions and hence the redox rhythm.

**Fig. 2.**
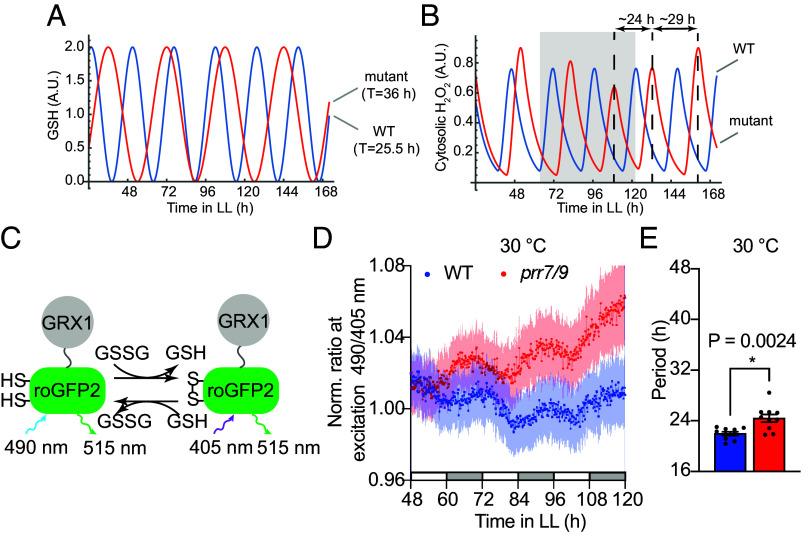
The dynamic interplay between the genetic clock and the redox rhythm. (*A* and *B*) A simplified mathematical model based on del Olmo et al. (13) for the coupling between the genetic clock and redox rhythm through glutathione oscillation. Reduced glutathione is driven at 25.5 h in WT (blue) or 36 h in a long-period mutant (red) by the genetic clock (*A*) and scavenges H_2_O_2_. In this model, cytosolic H_2_O_2_ represents the redox rhythm (*B*). The shaded region in (*B*) shows the characteristic transient phase shift of the redox rhythm in the long-period mutant and the later timepoints show gradual entrainment of the redox rhythm by the genetic clock. (*C*) Schematic of the Grx1-roGFP2 sensor system for in planta measurement of the glutathione redox potential. Oxidized and reduced forms of Grx1-roGFP2 are excited at 405 nm and 490 nm, respectively, and emissions for both are collected at 515 nm. The switch between these forms is mediated by Grx1 with GSH/GSSG as the coenzymes. (*D*) Temporal dynamics of normalized chloroplast-localized roGFP2 fluorescence ratio in plants grown under LL at 30 °C. n = 12 and n = 11 leaves for WT and *prr7/9*, respectively, from at least 2 independent transgenic lines. Individual dots are the means with shaded regions indicating the SEM. (*E*) Periods of rhythmic (RAE ≤ 0.7) individual leaves from (*D*) calculated using FFT-NLLS were compared using two-sided Student’s *t* test. All values are means ± SEM.

The regulation of the overall glutathione production by the genetic clock provides a possible mechanism for the genetic clock to entrain the redox rhythm. This interplay might also explain the altered phase of GSSG in *prr7 prr9* compared to WT. To test this hypothesis, we modified a previously proposed simple mathematical model for the redox rhythm (13) to introduce periodic forcing by a long-period, genetic clock-driven glutathione oscillation (Fig. 2*A*), which resulted in a similar transient phase shift for the redox rhythm (Fig. 2*B*).

To verify our findings in planta, we used the glutaredoxin (GRX)-fused redox-sensitive GFP (roGFP2) reporter system. roGFP2 can be excited by two different wavelengths of light depending on its oxidation state, allowing real-time ratiometric monitoring of glutathione redox potential (Fig. 2*C*). Since chloroplasts are the primary source of reducing power and energy in plants, we transformed roGFP2 variant localized to chloroplastic stroma (roGFP2_chl) (29) into WT and *prr7 prr9* mutant to monitor the redox rhythm in live plants. We used a custom-built fluorescence imaging chamber to perform time-lapse measurements of reduced and oxidized roGFP2_chl under LL at 30 °C, the temperature at which there is a large separation between the genetic clock and the redox rhythm periods (Fig. 1*H*). We used the normalized ratio of the roGFP2 fluorescence after excitation with 2 different wavelengths. We detected robust oscillations of the fluorescence ratio (Fig. 2*D*), indicating the viability of roGFP2 as an in vivo readout of the redox rhythm. The analysis using Biodare’s FFT-NLLS algorithm (30) of individual traces confirmed that, in agreement with the GSSG data (Fig. 1*H*), the roGFP2 ratio in *prr7 prr9* oscillated with a close-to-24 h period (Fig. 2*E*), instead of expected genetic clock period of ~36 h. A phase difference similar to the one in GSSG measurements was also observed (Figs. 1*F* and 2*D*), further indicating that these oscillations are endogenous and represent the redox rhythm as measured by GSSG.

### Transcriptional Targets of the Redox Rhythm Are Distinct from Those of the Genetic Clock Based on Periods.

**Fig. 3.**
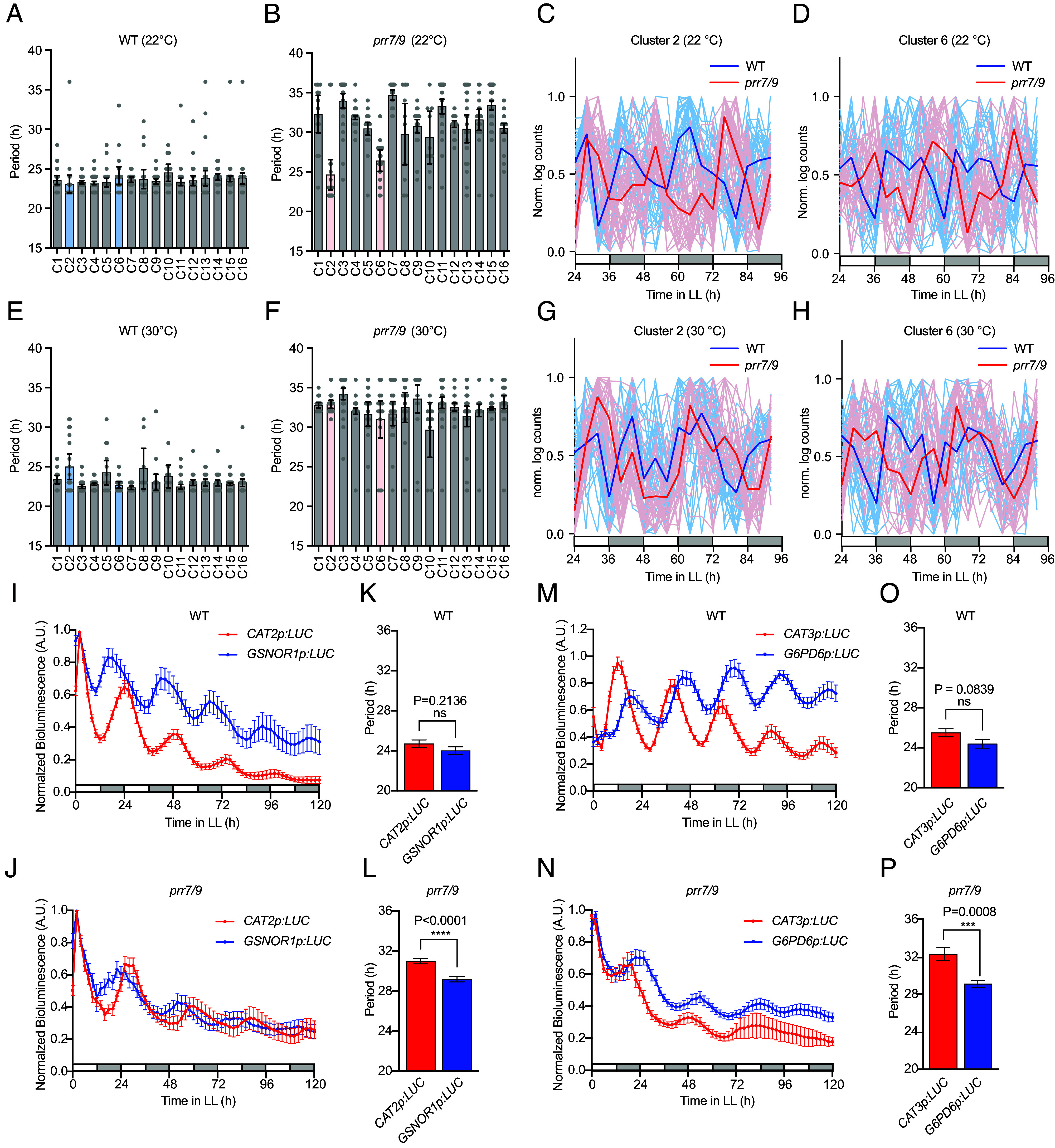
In *prr7 prr9*, two gene clusters of similar temporal expression patterns oscillate with periods distinct from those of the genetic clock. (*A* and *B*) Periods of oscillatory transcripts in WT (*A*) and *prr7 prr9* (*prr7/9*) (*B*) at 22 °C. Clusters were generated based on *k*-means analysis of expression signatures of all transcripts in the RASL-seq dataset from the *prr7/9* at 22 °C samples. For each cluster, periods of statistically significant (*P* < 0.05) oscillatory transcripts in WT and *prr7 prr9* were estimated using CosinorPY, with each dot representing the period of a single transcript. Clusters of interest, C2 and C6, are colored blue (*A*) and red (*B*), respectively. (*C* and *D*) Normalized expression levels of all genes in C2 (*C*) and C6 (*D*) in WT and *prr7/9* at 22 °C. Dark lines represent the means of each cluster, while light lines represent the expression pattern of the means of individual genes in the clusters. (*E*–*H*) The data for the same clusters as in (*A*–*D*) but at 30 °C. (*I* and *J*) Normalized bioluminescence traces for shoot apices of all independent T1 transformants from a single experiment of *CAT2p:LUC* and *GSNOR1p:LUC* in WT with n = 16 and n = 8, respectively (*I*) or in *prr7/9* with n = 13 and n = 10, respectively (*J*). (*K* and *L*) Period estimation by FFT-NLLS of all rhythmic (RAE ≤ 0.7) T1 transformants from three separate experiments, combined using linear mixed-effect model (LMM), for *CAT2p:LUC* and *GSNOR1p:LUC* in WT with n = 18 and n = 18, respectively (*K*) and in *prr7/9* with n = 24 and n =28, respectively (*L*). (*M* and *N*) Normalized bioluminescence traces for shoot apices of all independent T1 transformants from a single experiment of *CAT3p:LUC* and *G6PD6p:LUC* in WT with n = 9 and n = 8, respectively (*M*) or in *prr7/9* with n = 10 and n = 13, respectively (*N*). (*O* and *P*) Period estimation by FFT-NLLS of all rhythmic (RAE ≤ 0.7) T1 transformants from two separate experiments, combined using LMM, for *CAT3p:LUC* and *G6PD6p:LUC* in WT with n = 19 and n = 12, respectively (*O*) and in *prr7/9* with n = 10 and n =19 transformants, respectively (*P*). Periods were compared using two-tailed Student’s *t* test; ns, not significant; A.U., Arbitrary Units. Values are means ± 95% CI for (*A, B*, *E*, and *F*) and means ± SEM for (*I–P*).

The distinct oscillation periods for the redox rhythm and the genetic clock in the *prr7 prr9* mutant provided an opportunity to identify their specific transcriptional targets via clustering. We designed a two-step strategy to identify oscillatory genes driven by the redox rhythm. Since we are interested in not only period length of the target genes, but also their coexpression in conferring a specific physiological function, we first clustered the transcripts according to their normalized temporal signature using *k*-means clustering of the circadian time course RASL-seq data in the *prr7 prr9* mutant at 22 °C. After testing different k values, we chose k = 16 to accommodate all possible clusters for the genes oscillating with the genetic clock period of approximately 32 h (8 points for each of the possible phases given by the time resolution of 4 h, multiplied by 2 to account for positive and negative skew). For genes in each coexpression cluster defined using the *prr7 prr9* at 22 °C data, the second step was to identify rhythmic transcripts in WT and the *prr7 prr9* mutant at 22 °C and 30 °C using the CosinorPY package (31) with the significance cutoff of *P* < 0.05. While a close-to-24 h period was observed across all coexpression clusters in the WT at 22 °C, larger period length variations were found in the *prr7 prr9* mutant with majority of the gene clusters oscillating with the expected genetic clock period of ~32 h (Fig. 3 *A* and *B*). Interestingly, clusters 2 and 6 (C2 and C6, Dataset S2) predominantly contained oscillatory genes (shown as dots) with periods close to that in WT. Since the output of *k*-means clustering can vary depending on the initial seed and the number of clusters, we also used a deterministic hierarchical algorithm to analyze the RASL-seq data, which largely recapitulated the results of the *k*-means clustering (*SI Appendix,* Fig. S1).

To rule out the possibility that the observed period-length separation of the circadian genes in *prr7 prr9* was caused by tissue-specificity (32–34), we performed further analysis on the RASL-seq data generated using homogenized leaf tissues by grouping genes with known tissue specificity versus those without (35) and found that C2 and C6 genes did not originate from a single tissue and were still separated from others with their distinctly shorter periods across all groups (*SI Appendix,* Fig. S2*A*), indicating that period separation of C2 and C6 is unlikely due to a tissue-specific effect. We also examined two genetic clock-driven genes *CHLOROPHYLL A/B-BINDING PROTEIN 2* (*CAB2*) and *CATALASE 3* (*CAT3*) which were reported to have a ~2 h-difference in period length in WT (36) and found that this difference persisted in *prr7 prr9*, but with both periods >32 h (*SI Appendix,* Fig. S2*B*), unlike those of genes in C2 and C6. Finally, we used another package, MetaCycle (37), to analyze our RASL-seq data by a combination of two additional algorithms, JTK_CYCLE and Lomb–Scargle periodogram, to independently verify our results from CosinorPY analysis. After analyzing statistically significant oscillations (*P* < 0.05), we again found C2 and C6 to oscillate with a shorter period in the *prr7 prr9* mutant (*SI Appendix,* Fig. S2 *C* and *D*). Notably, oscillations for genes in both C2 and C6 also showed altered phase in *prr7 prr9* compared to WT at 22 °C, resembling that of GSSG (Figs. 1*E* and 3 *C* and *D*), consistent with these genes being regulated by the redox rhythm.

Surprisingly, at 30 °C, virtually none of the genes in C2 and C6 kept the shorter period (Fig. 3 *E*–*H* and *SI Appendix,* Fig. S2 *E* and *F*). At this elevated temperature, these genes appeared to be driven by the genetic clock. Therefore, the C2 and C6 genes can be regulated by both the redox rhythm and the genetic clock, with the redox rhythm playing the primary role under the normal temperature and the genetic clock dominating at elevated temperatures (Fig. 3 *A*–*H*). Among the genes studied in our RASL-seq, a single gene from C13, the key flowering time regulator *FLOWERING LOCUS T* (*FT*) (38) reliably oscillated with a shorter period under both temperatures (*SI Appendix,* Fig. S3 *A* and *B*), in agreement with the near WT redox rhythm period observed in the *prr7 prr9* mutant (Figs. 1 *G* and *H* and 2 *D* and *E*). These results imply that at 30 °C, while the C2 and C6 genes are delinked from the redox rhythm, *FT,* and perhaps other flowering time genes not present in our RASL-seq gene pool, is still under the circadian redox rhythm regulation, likely through a different signaling pathway.

Since time-course experiments may lead to inaccurate period estimation due to the low time resolution and plant sample variations, we next constructed bioluminescence reporter lines to verify our findings. Based on previous publications (12, 22), we chose the promoter of *CATALASE 2* (*CAT2*), a morning-phased output gene of the genetic clock (*SI Appendix,* Fig. S3 *C* and *D*), to drive the luciferase reporter. To make a reporter for the redox rhythm output, we chose, from C6, the promoter of *S-NITROSOGLUTATHIONE REDUCTASE 1* (*GSNOR1*), which is the key regulator of S-nitrosothiol (SNO) levels in plants (39) (Fig. 1*A*). Besides its robust oscillation detected in our RASL-seq data (*SI Appendix,* Fig. S3 *E* and *F*), the dependence of GSNOR1 activity on NADH and GSH (40) further connects the gene to the redox rhythm. *CAT2p:LUC* and *GSNOR1p:LUC* constructs were transformed into both WT and the *prr7 prr9* backgrounds and all independent T1 lines were imaged to minimize insertion position effects. Since the leaf age may affect the period of the circadian clock (41) within the same plant, and to avoid reported tissue-specific effects (32–34), we quantified the luminescence of the shoot apex, whose circadian rhythms are robust and highly synchronized (42). The period length was then compared for all rhythmic lines [relative amplitude error (RAE) less than or equal to 0.7] across three independent experiments. In agreement with the RASL-seq data, significant differences in period lengths of *CAT2p:LUC* and *GSNOR1p:LUC* oscillations were detected in *prr7 prr9*, but not in WT at 22 °C (Fig. 3 *I*–*L*). Interestingly, for the last oscillation cycle, *GSNOR1p:LUC* oscillation period appeared to lengthen to match the *CAT2p:LUC* oscillation. This is likely due to the entrainment of the redox rhythm by the genetic clock through rhythmic transcription of *GSH1* (Fig. 1 *L*–*N*). In support of this hypothesis, we recapitulated similar results during the later timepoints of the mathematical model (Fig. 2*B*), indicating the transient nature of the period separation between the redox rhythm and the genetic clock in the *prr7 prr9* mutant. To ascertain that the period separation is not limited to this pair of genes, we tested another pair of reporters. *CATALASE 3* (*CAT3*) was chosen as an evening-phased genetic clock output (12) to ensure that both morning- and evening-phased genetic clock outputs were separated from the redox rhythm. For the redox rhythm, the *GLUCOSE-6-PHOSPHATE DEHYDROGENASE 6* (*G6PD6*) gene encoding an enzyme from the oxidative Pentose Phosphate Pathway (PPP) (43) (Fig. 1*A*) was chosen because PPP is a hub for the interplay between the redox rhythm and the genetic clock in mammals (14, 15) and the *G6PD6* gene represents a different redox-regulated gene cluster (C2 in Fig. 3*B*). As with the previous pair of reporters, we found that *G6PD6p:LUC* oscillated with a significantly shorter period than *CAT3p:LUC* (Fig. 3 *M*–*P*) in *prr7 prr9,* but not in WT. Therefore, we observed the coexistence of the redox and genetic circadian rhythms within the same tissue type (shoot apical meristem) based on the distinct periods of their output genes (Fig. 3 *L* and *P*), further confirming the results from the RASL-seq, glutathione, and roGFP2 measurements (Figs.1 *G* and *H*, 2*E*, and 3*B*).

### Redox Rhythm Regulates Immune-Induced Programmed Cell Death.

**Fig. 4.**
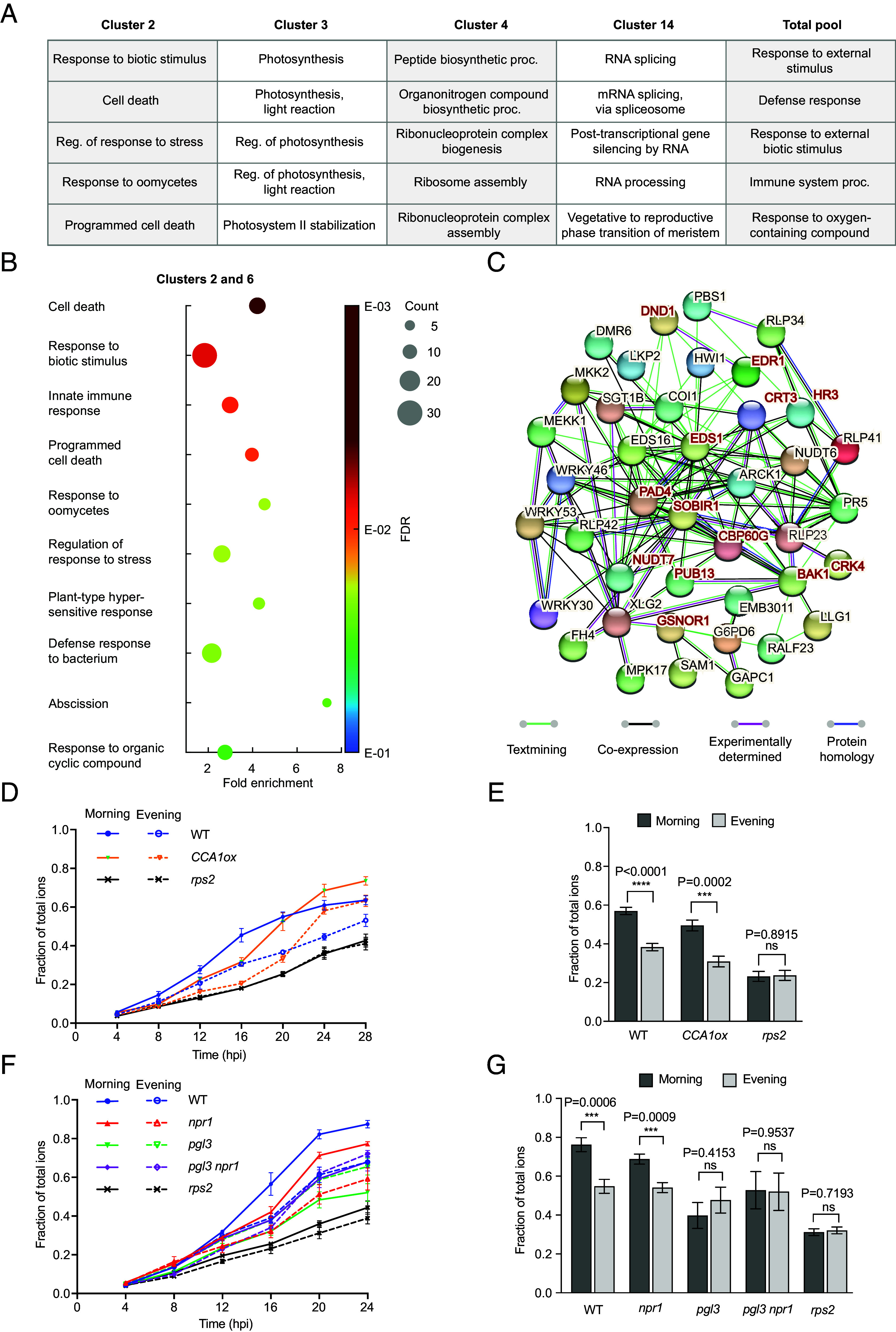
Redox rhythm regulates immune-induced PCD. (*A*) Top 5 GO terms based on the false discovery rate (FDR) for all individual clusters with significant enrichment using the total RASL-seq gene pool (~700 genes) as the background, or total RASL-seq gene pool compared to the entire *Arabidopsis* genome (last column). (*B*) GO terms for combined C2 and C6 genes, sorted by FDR. (*C*) StringDB analysis for C2 and C6 genes using text mining, coexpression, experimental evidence, and protein homology. Interaction score was set to 0.400. All genes belonging to the top GO term, cell death, are present and labeled in red letters. (*D* and *E*) Time-course of ion leakage (a measure of cell death). After infiltration with *Psm* ES4326/*avrRpt2* in WT, *CCA1ox*, and *rps2*, ion leakage was estimated by measuring conductivity at each timepoint normalized to conductivity of total ions for each sample (*D*). Analysis of normalized conductivity at 20 hours post infiltration (hpi) combined from three separate experiments using LMM (*E*). Two-tailed Student’s *t* test, ns, not significant. (*F* and *G*) Time-course of ion leakage measured as in (*D*) in WT, *npr1*, *pgl3*, *pgl3 npr1*, and *rps2* (*F*). Normalized conductivity at 20 hpi combined from three separate experiments (*G*) was analyzed as in (*E*). n = 3 biological replicates per timepoint for (*D* and *F*), and n = 9 biological replicates pooled from three separate experiments for (*E* and *G*). All values are means ± SEM.

To test whether genes with similar temporal patterns (i.e., clusters) have a common biological function, gene ontology (GO) enrichment analysis was performed, with the RASL-seq probe set as the background to eliminate the bias stemming from using a limited gene pool. Remarkably, even in this probe set mostly containing genes involved in different immune responses (last column in Fig. 4*A*), the C2 genes were found to be significantly enriched with those involved in the regulation of immunity and cell death (predominantly immune-induced PCD, known as hypersensitive response in plants), while C6 had no significant enrichment possibly due to the low number of genes in the cluster. Other clusters with significant GO term enrichments had photosynthesis (C3)-, growth (C4)-, and messenger RNA (mRNA) processing (C14)-related genes (Fig. 4*A*). When the redox rhythm target genes (C2 and C6) were combined for GO enrichment analysis, immune-related GO terms were found again, similar to C2 alone, though with the cell death becoming the top GO term (Fig. 4*B*). Further STRING (Search Tool for the Retrieval of Interacting Genes/Proteins) analysis uncovered a highly interconnected network, with known immune-induced cell death regulator genes such as *SUPPRESSOR OF BIR1-1* (*SOBIR1*) (44), *PHYTOALEXIN DEFICIENT 4* (*PAD4*) and *ENHANCED DISEASE SUSCEPTIBILITY 1* (*EDS1*) (45) in hub positions (Fig. 4*C*). Moreover, in agreement with our clustering analysis based on temporal expression signatures, STRING detected a high degree of coexpression of genes in clusters 2 and 6 according to their publicly available data (black lines in Fig. 4*C*). Moreover, analysis of only those genes showing statistically significant oscillations (*P* < 0.05) (Dataset S3), produced similar results (*SI Appendix,* Fig. S4). Based on the identities of C2 and C6 genes, we hypothesized that the circadian redox rhythm regulates PCD during the effector-triggered immunity (ETI), which occurs when the presence of a pathogen effector is recognized by its cognate nucleotide-binding site, leucine-rich repeat (NB-LRR) receptor in the plant host. This immune response is known to be associated with ROS burst (46) and significant increase in ATP levels (47, 48). It is also known that glutathione depletion severely compromises PCD (25). Moreover, while the time-of-day circadian sensitivity to ETI-triggered PCD has been reported previously (49), no mechanism has been proposed for this regulation. Remarkably, consistent with the delinking of C2 and C6 genes from the redox rhythm at 30 °C (Fig. 3 *F*–*H*), ETI-mediated PCD is well known to be inhibited at elevated temperatures (50).

To test the circadian control of PCD by the redox rhythm, we used the bacterial pathogen *Pseudomonas syringae* pv. *maculicola* ES4326 carrying the effector gene *avrRpt2* (*Psm* ES4326/*avrRpt2*) to infect the WT, genetic clock-defective *CIRCADIAN CLOCK ASSOCIATED 1* overexpressing (*CCA1ox*) line (51) and the mutant lacking the NB-LRR receptor for avrRpt2, *resistant to p. syringae 2* (*rps2*). Based on a previous study, which showed that, for two consecutive circadian cycles under constant light (LL), ETI-induced PCD was circadian-gated toward the morning in WT (49), we examined the presence or absence of this time-of-day regulation of PCD by performing ETI-induced cell death assays in the subjective morning and subjective evening after 2 d of growth under constant light (LL) to eliminate any transient oscillations in *CCA1ox* (*SI Appendix,* Fig. S5 *A* and *B*). In agreement with our hypothesis, both WT and *CCA1ox* showed stronger ETI-associated PCD in the subjective morning than in the subjective evening (Fig. 4 *D* and *E*), even though *CCA1ox* has been previously reported to be compromised in the time-of-day-sensitive basal resistance to the bacterial pathogen without the avrRpt2 effector (52). In contrast to the genetic clock-defective *CCA1ox*, the mutant of *6-PHOSPHOGLUCONOLACTONASE 3* (*PGL3*), encoding a key enzyme in the oxidative section of the plastidic PPP (Fig. 1*A*), which has markedly altered glutathione levels and constitutively enhanced resistance (53), had decreased PCD and lost the time-of-day sensitivity to ETI induction (Fig. 4 *F* and *G*). This result is in accordance with the finding that a functional PPP is required in the anucleate human red blood cells for maintaining the redox rhythm (10). Since the enhanced basal resistance phenotype of *pgl3* is dependent on the constitutively activated NONEXPRESSOR OF PATHOGENESIS-RELATED GENES 1 (NPR1) (53), whose nuclear translocation is redox rhythm-sensitive (12), we also tested the *npr1* single and the *pgl3 npr1* double mutants for ETI-induced PCD. We found that the *npr1* single mutant still had time-of-day sensitivity to immune-triggered PCD, but when combined with *pgl3,* it could not rescue the PCD phenotype of *pgl3* (Fig. 4 *F* and *G*), indicating that the lack of circadian PCD in *pgl3* is not due to constitutive activation of NPR1-mediated resistance. Finally, we used the *toc1 lhy cca1* core clock gene triple mutant (54) as an alternative genetic clock-defective mutant and the *pad2* mutant of *GSH1* (25) (Fig. 1*A*) as an alternative redox mutant. We observed that *toc1 lhy cca1* still exhibited significant time-of-day difference in PCD levels, in contrast to *pad2* (*SI Appendix,* Fig. S5 *C* and *D*), further confirming that circadian PCD is driven by the redox rhythm, not the genetic clock.

### Redox Rhythm Regulates PCD through the Jasmonic Acid (JA)/Ethylene (ET) Defense Pathway.

NPR1 has previously been shown to be involved in salicylic acid (SA)-mediated inhibition of ETI-induced PCD (55) and gating of SA-target gene expression through an increase in the amplitude of the genetic clock (12). However, the circadian gating of PCD observed in the *npr1* mutant suggests that this redox rhythm-mediated phenomenon is through an SA/NPR1-independent pathway. It is known that besides SA, other defense hormones, such as JA and ET, also contribute to ETI (56, 57). Among the target genes regulated by the redox rhythm, both EDS1 and PAD4 in C2 (Fig. 4*C* and Dataset S2) are critical regulators of SA and JA crosstalk during ETI (58), even though they are not required for the function of the coiled-coil (CC) class of NB-LRRs (45) used in this study. Furthermore, C2 and C6 also include regulators of immune responses mediated by JA and ET (Fig. 4*C* and Dataset S2). GSNOR1 in C6 is known to both regulate PCD (39, 59) and promote JA-induced defense against herbivores in tobacco (60). SOBIR1 and RECEPTOR LIKE PROTEINs (RLPs) in C2 are involved in PCD (61, 62) and JA/ET-mediated defense against necrotrophic fungi (63). These findings suggest that the circadian redox rhythm may regulate PCD through the JA and/or the ET defense pathways.

**Fig. 5.**
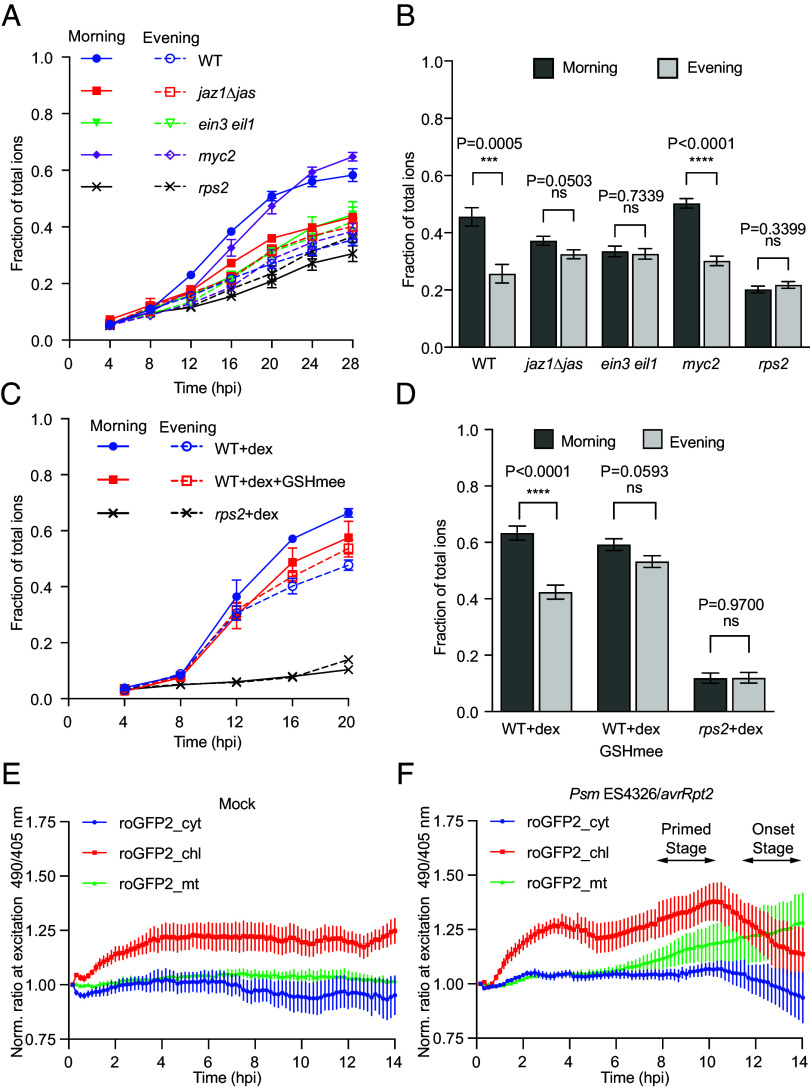
Redox rhythm regulates PCD through the JA/ET defense hormone pathway. (*A* and *B*) Time-course of ion leakage after infiltration with *Psm* ES4326/*avrRpt2* in WT, *jaz1Δjas*, *ein3 eil1*, *myc2*, and *rps2*. Conductivity was normalized to total ions for each sample (*A*). Analysis of normalized conductivity at 20 hpi combined from three separate experiments using a LMM (*B*). Two-tailed Student’s *t* test, ns, not significant. (*C* and *D*) Time-course of ion leakage measured as in (*A*) of WT and *rps2* plants carrying *Dex:avrRpt2* after treatment with dex or dex + GSHmee (*C*). Analysis of normalized conductivity at 20 hpi from three separate experiments (*D*) was carried out as in (*B*). n = 3 biological replicates per timepoint for (*A* and *C*), and n = 9 biological replicates pooled from three separate experiments for (*B* and *D*). (*E* and *F*) Temporal dynamics of normalized roGFP2 fluorescence (515 nm) ratio after excitations at 490 nm and 405 nm in the cytoplasm (roGFP2_cyt), chloroplasts (roGFP2_chl), and mitochondria (roGFP2_mt) in mock (*E*) and *Psm* ES4326/*avrRpt2*-infiltrated (*F*) leaves before tissue collapse. The ratios were normalized to values of the initial timepoint to monitor the dynamics (29). n = 4 leaves for each condition/genotype. All values are means ± SEM.

The JA defense pathway is normally suppressed by the JASMONATE-ZIM-DOMAIN PROTEIN (JAZ) proteins which are degraded during ETI (56). Downstream of JAZ, the JA pathway contains two mutually antagonistic branches: the JA-only branch, with MYC2 as the central transcription factor (TF) promoting defense against herbivores, and the JA/ET-shared branch, with ETHYLENE-INSENSITIVE3 (EIN3) and ETHYLENE-INSENSITIVE3-LIKE 1 (EIL1) as key TFs protecting against necrotrophic pathogens (64). To determine which of the JA signaling branches is involved in the circadian control of PCD, we used the noninducible *jaz1Δjas* mutant expressing a JAZ1 protein lacking the Jas degradation domain (65) and key TF mutants *myc2* and *ein3 eil1* to assess PCD after infiltration with *Psm* ES4326/*avrRpt2*. We found that while both the *jaz1Δjas* and *ein3 eil1* mutants lost their time-of-day sensitivity to ETI-triggered PCD, *myc2* retained it (Fig. 5 *A* and *B*), indicating that an intact JA/ET branch is necessary for circadian regulation of ETI-associated PCD. Similar results were obtained for WT and *ein3 eil1* when comparing PCD levels after infection at subjective evening of day 2 and at subjective morning of day 3 under LL, indicating that the circadian redox rhythm, rather than prolonged exposure to constant light, is responsible for this regulation (*SI Appendix,* Fig. S5 *E* and *F*).

To further demonstrate the role of the redox rhythm in the circadian regulation of PCD, we used the cell permeable GSH derivative, glutathione monoethyl ester (GSHmee), to perturb the cellular redox rhythm. GSHmee treatment has previously been shown to disrupt the redox rhythm while reinforcing the genetic clock through the activity of NPR1 (12). To exclude a possible influence of GSHmee on the growth of the pathogen, we used the transgenic plants that express the bacterial effector avrRpt2 in response to dexamethasone (dex) treatment to induce PCD. We found that while the treatment in the subjective morning triggered a stronger PCD than in the subjective evening, cotreatment with GSHmee abolished this difference (Fig. 5 *C* and *D*). This further links cellular glutathione levels to the time-of-day sensitivity of ETI-induced PCD.

Finally, we used the roGFP2_chl with additional roGFP2 variants localized to the cytoplasm (roGFP2_cyt) and the mitochondrial matrix (roGFP2_mt) (29) to evaluate the subcellular compartment-specific redox contribution to ETI-mediated PCD. Whole-plant time-lapse imaging (Fig. 5 *E* and *F*) and subsequent confocal microscopy (*SI Appendix,* Fig. S6) showed that upon ETI-induction, chloroplasts and mitochondria first became strongly reduced at the “primed stage,” while cytoplasmic redox remained unchanged compared to mock, even though ETI signaling is associated with ROS production through plasma membrane-associated NADPH oxidase activities (46). However, immediately before the onset of PCD (“onset stage”), we observed a switch to a more oxidized state in chloroplasts and the cytosol, but not in mitochondria. This observation aligns with the recently proposed hypothesis that during ETI, chloroplast stroma is first overreduced due to removal of RuBisCO through autophagy (66), which ultimately helps trigger the production of ROS, making chloroplasts the major source of cellular oxidation during ETI-induced PCD (67).

## Discussion

**Fig. 6.**
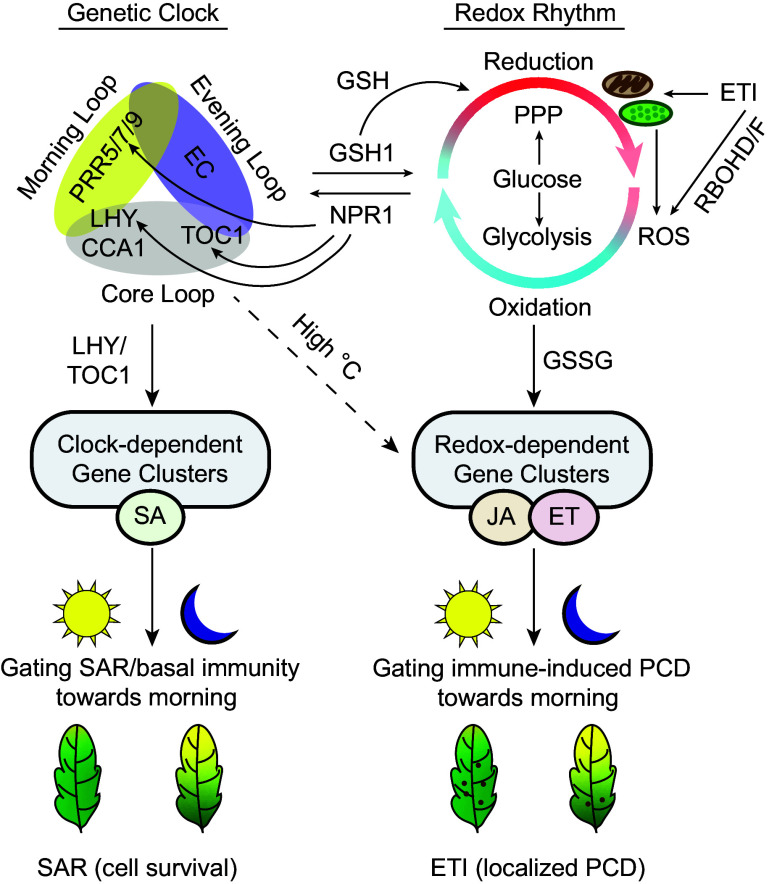
The model of circadian regulation of immunity in plants. Genetic clock and redox rhythm are coupled through proteins such as NPR1 and GSH1, which are known to regulate genetic clock gene expression (12) and GSH biosynthesis (26), respectively. The genetic clock controls basal resistance/SAR through TFs such as LHY and TOC1 (12), while the redox rhythm generated through metabolic pathways such as glycolysis, PPP, and photosynthetic/mitochondrial activities in plants controls distinct transcriptional targets, including those involved in ETI-induced PCD. Upon ETI induction, ROS is generated by NADPH oxidases RBOHD/F (46) for signaling while ATP (48) and reducing power are provided through reprogramming of chloroplast and mitochondrial activities. Subsequent accumulation of ROS in chloroplasts helps trigger PCD which is enhanced in the morning through the JA/ET defense pathway. Under high temperature, the redox-dependent PCD-gene clusters shift to being controlled by the genetic clock through an unknown mechanism (dashed line). EC, evening complex.

In recent years, there has been increasing evidence showing that various autonomous oscillators coexist with canonical biological oscillatory systems. For example, the central Cyclin/Cyclin-dependent kinase oscillator was recently shown to phase-lock an autonomous suboscillator to coordinate organelle biogenesis (68). However, the physiological output, and therefore, the biological significance of the redox rhythm remained elusive for more than a decade after it was first discovered (11). Our study firmly establishes ETI-triggered PCD as a biological process specifically regulated by the circadian redox rhythm through the JA/ET defense signaling pathway and demonstrates its biological significance in regulating plant cell fate upon pathogen challenge (Fig. 6). This circadian control of PCD is distinct from the genetic clock-regulated expression of antimicrobial genes mediated by the defense hormone SA and signaling protein NPR1 in promoting cell survival in response to pathogen challenge (12, 55) (Fig. 6). Despite their distinguishable roles in regulating different plant immune responses, redox rhythm and genetic clock are intrinsically intertwined through the activities of proteins, such as NPR1 which responds to redox perturbations by enhancing the amplitude of the genetic clock (12). In the reverse direction, the genetic clock controls the production of redox molecules, such as glutathione, through transcription of genes, such as *GSH1* (Fig. 1 *A* and *I*–*N*). The use of the long-period mutant *prr7 prr9* allowed us to disentangle the two oscillatory systems, at least transiently, under LL. This dynamic interplay between the two oscillations in *prr7 prr9* could explain the phase shift of GSSG in the mutant compared to WT and the possible entrainment of the redox rhythm by the genetic clock (Figs. 1 *E* and *F*, 2 *B* and *D*, and 3*J*). It would be interesting to study the dynamics of the interplay between the redox rhythm and the genetic clock in other clock period mutants. If this transient separation is achievable only in the *prr7 prr9* mutant that would imply a direct role for PRR7 and/or PRR9 in coupling the circadian redox rhythm to the genetic clock.

The regulation of ETI-associated PCD by the redox oscillation, rather than the genetic clock, might be due to the intense energy requirement for this immune response which is known to involve the expenditure of ATP (47, 48) and transcriptional and translational reprogramming (69). The ATP level increase (48) is likely correlated with the reduced state of chloroplasts and mitochondria observed in the primed stage of ETI (Fig. 5 *E* and *F* and *SI Appendix,* Fig. S6) due to the interconnection between reducing power and ATP synthesis. Gating PCD toward the morning would allow the response to coincide with the production peaks of energy (ATP) (70), reducing power (GSH) (Fig. 1 *I* and *L*), as well as JA production which also peaks in mid-morning (71). Based on literature, redox rhythm is connected to both the PPP and glycolysis (10), and the shifts of metabolic flux between these pathways might lie at the core of the redox rhythm-mediated regulation of cellular responses. The abolishment of circadian PCD in *pgl3* (a chloroplast localized PPP enzyme) mutants (Fig. 4 *F* and *G*) supports this hypothesis. Interestingly, while the origin of the redox rhythm had initially been hypothesized to be connected to the great oxidation event (GOE) due to the appearance of oxygenic photosynthesis (9), a more recent study suggests that evolution of antioxidant proteins such as PRXs significantly preceded GOE (72). Since the major function of PRXs consists of scavenging the ROS produced in chloroplasts and mitochondria as a byproduct of their activities (73, 74), it is possible that the activities of photosynthetic and mitochondrial electron transport chains are the origin and the ultimate driver of the redox rhythm.

Based on the results of this study, we hypothesize that with redox rhythm serving as a sensitive signaling hub, organisms gain greater flexibility in minimizing metabolic conflict caused by pathogen challenge. This hypothesis is reinforced by the lack of increased morning PCD and lower overall cell death in the glutathione-deficient *pad2* mutant (*SI Appendix,* Fig. S5 *C* and *D*), and increased evening PCD with the addition of reducing power through GSHmee supplementation (Fig. 5 *C* and *D*). However, the role of GSH in promoting PCD is nuanced. While the lack of GSH in *pad2* does inhibit PCD, one of the primary ways the cells utilize GSH is to detoxify ROS through the ascorbate-glutathione cycle (Fig. 1*A*). This includes the ROS produced during PCD. Therefore, excessive levels of GSH can also suppress PCD (75). Conversely, while the direct application of ascorbate does not significantly affect PCD (75), ascorbic acid-deficient mutants exhibit spontaneous cell death (76).

The scope of this study was defined by the specific pool of probes used in the RASL-seq and the measurement of a single redox pair of GSH/GSSG due to our research focus and technical limitations. However, the successful use of roGFP2 to monitor the redox rhythm in planta (Fig. 2 *D* and *E*) presents a great opportunity to significantly expand the scope of such studies to other organisms. In contrast to the labor-intensive time-course measurements of PRX-SO_2/3_ or redox couples, the roGFP sensors allow observation of redox changes in different subcellular compartments in real time.

The incidental detection of the flowering time gene *FT* which oscillates with a shorter period in the long period *prr7 prr9* mutant suggests the presence of other target genes and physiological processes that are controlled by the redox rhythm. Given that the expression of *FT* is tied to the daylight length and the cellular energy reserves represented by sugar (trehalose) levels (77), one might speculate that the redox rhythm is the natural hub tying photoperiodic behaviors to the energy metabolism. This might also explain why *FT* keeps shorter period in *prr7 prr9* even at high temperatures (i.e., still likely driven by the redox rhythm), which, in contrast to PCD, do not inhibit flowering (78). It is intriguing that another flowering time regulator, FLOWERING LOCUS K HOMOLOGY DOMAIN (FLK), was recently shown to control plant immunity and cell death (79). An investigation of the possible connections between redox, flowering, and plant immunity is an intriguing future direction.

The control of C2 and C6 genes by the genetic clock at 30 °C is a remarkable phenomenon that requires further investigation. It is possible that the delinking of these genes from the redox rhythm under elevated temperatures is associated with the suppression of ETI-PCD under those conditions (50), explaining its significance. Besides ETI-associated PCD, C2 and C6 also regulate other cellular functions such as basal immunity (Fig. 4*B*), explaining the need for the secondary layer of circadian control by the genetic clock. Likely TFs responsible for such regulation include EIN3 and EIL1, involved in redox rhythm-mediated gating of PCD (Fig. 5 *A* and *B*), each containing eleven potentially redox-sensitive cysteines, and exhibiting circadian oscillations at the transcript level (80). Thus, while their transcript levels are controlled by the genetic clock, their activity could be determined by the cellular redox (rhythm) in a temperature-dependent manner. A more comprehensive whole transcriptome/metabolome analysis of genetic clock, redox, and JA/ET mutants as well as protein studies of EIN3/EIL1 in the future would not only uncover the mechanisms by which redox rhythm gates PCD via the JA/ET pathway but also reveal possible new outputs of the redox rhythm in regulating plant physiology. Our study suggests that regulation of major incidental energy-consuming events, such as immune-induced PCD, might be the universal reason for the conservation of the ancient redox rhythm across all lineages of life during evolution (9) even after the subsequent emergence of genetic clocks.

## Methods Summary

### Materials.

All *Arabidopsis thaliana* WT, mutants, and transgenic plants used in this study were in the Columbia-0 ecotype background. *Psm* ES4326/*avrRpt2* was used for PCD assays and *Agrobacterium tumefaciens* strain GV3101 was used for plant transformation. The plasmids for the luciferase reporter lines were constructed using the Gateway Technology (Thermo Fisher Scientific).

### RNA Extraction, RASL-seq, and RT-qPCR.

RNA extraction was performed using TRIZOL (Ambion). Probe annealing, ligation, and library construction for RASL-seq was performed as described in ref. 81. For RT-qPCR, *UBIQUITIN 5* (*UBQ5*) mRNA was used for normalization. Primer sequences are provided in *SI Appendix,* Table S1.

### RASL-seq Time-Course Data Clustering and Period Estimation.

The traces for the RASL-seq time-course data were detrended and normalized to ensure that the dominant signature of clustering was the oscillatory pattern. Then, *k-*means clustering of the genes was performed based on their temporal signature using MATLAB (R2022a) while the hierarchical clustering was done using http://www.bioinformatics.com.cn/srplot. The period distribution for each cluster was obtained using either CosinorPY (v3.1) (31) or MetaCycle (37). For individual genes as well as total glutathione and GSSG, harmonic regression was performed using GraphPad Prism 9 as described previously (12).

### Glutathione Measurements.

Glutathione extraction using HCl and colorimetric measurements using the Victor X5 plate reader (PerkinElmer) were performed based on the method described in ref. 82.

### Mathematical Modeling of Redox Rhythm Phase Shift in a Long-Period Mutant.

The 6-ODE mathematical model by del Olmo et al. (13) was modified using Mathematica (v13.3) by introducing a term for oscillating reduced glutathione scavenging H_2_O_2_:

$$-g\times \cos\left(\frac{2\pi t}{T}\right),$$

where *g* is the coupling constant (equal to 0.06 for Fig. 2*A*) and T is the period.

### Conductivity Assay for Measuring PCD.

To induce PCD, either *Psm* ES4326/*avrRpt2* (OD_600 nm_ = 0.02) or solution of 0.25 µM of dex and 0.01% Silwet L-77 (Plantmedia) with or without 30 µM GSHmee (Sigma) was used. Conductivity was measured using an Orion Star series meter (Thermo Scientific).

### Imaging and Analysis.

For luminescence, the plants were imaged using a custom-built chamber as described previously (22) using PIXIS 2048B camera. For fluorescence, a custom-built fluorescence chamber was used with excitation provided by CoolLED pE-4000 system and emission using the Andor iXon Ultra 888 EMCCD Camera. For microscopy, the imaging was done with the Zeiss 880 airyscan inverted microscope. All images were analyzed using ImageJ.

### Statistical Analysis and Data Processing.

All statistical analyses and harmonic regression were done using GraphPad Prism 9. Data processing was done in MATLAB (R2022a) or R (v4.1.2). GO term analysis was performed using ShinyGO (v0.77) (83). In the graphs, asterisks indicate statistical significance reflecting the *P* values (*P < 0.05, **P < 0.01, ***P < 0.001, ****P < 0.0001, and ns, not significant). Unless specified, experiments were repeated at least three times with similar results.

## Supplementary Material

Appendix 01 (PDF)

Dataset S01 (XLSX)

Dataset S02 (XLSX)

Dataset S03 (XLSX)

## Data Availability

Data are available in Datasets S1–S3 and Biodare repository (84–86). All study data are included in the article and/or supporting information.
