# Supplementary material for: The redox rhythm gates immune-induced cell death distinctly from the genetic clock
Source: Proc Natl Acad Sci U S A. 2025 Sep 10;122(37):e2519251122. doi: 10.1073/pnas.2519251122 (PMC12452835; doi:10.1073/pnas.2519251122)
Supplement: Supplementary file 1 — Appendix 01 (PDF) [file pnas.2519251122.sapp.pdf]

PNAS

Supporting Information for

Redox rhythm gates immune-induced cell death distinctly from genetic clock

Sargis Karapetyan[a,b], Musoki Mwimba[a,b], Tianyuan Chen[a,b], Zhujun Yao[a], and Xinnian Dong[a,b,1]

[a] Department of Biology, Box 90338, Duke University, Durham, NC 27708, USA

[b] Howard Hughes Medical Institute, Duke University, Durham, NC 27708, USA

[1]To whom the correspondence may be addressed. Email: xdong@duke.edu

This PDF file includes:

Materials and Methods
Figures S1 to S6
Table S1
SI References

Other supporting materials for this manuscript include the following:

Datasets S1 to S3

Materials and Methods

Plant growth

All *Arabidopsis thaliana* wild-type (WT), mutants, and transgenic plants used in this study were in the Columbia-0 (Col-0) ecotype background. Mutants *prr7 prr9* (1), *toc1 lhy cca1* (1), *CCA1ox* (2), *rps2* (3), *npr1* (3), and *jaz1Δjas* (4), as well as dex-inducible avrRpt2 transgenic lines (3) were described previously. Seeds for *pad2* (5) were obtained from the Arabidopsis Biological Resource Center. The seeds for *pgl3* and *pgl3 npr1* mutants (6) were generously provided by Dr. Z. Mou, *ein3 eil1* (7) and *myc2* (8) by Dr. H. Guo. Transformation into *Arabidopsis* using the floral dip method and antibiotic selection were performed according to Harrison et al. (9).

The plants (except T1 transformants) were grown on soil (Metromix 360 or Metromix 830) for 4-5 weeks (5-6 weeks for *prr7 prr9* and *CCA1ox*) in Percival chambers under 12 h/12 h light/dark cycles with daytime light intensity of 80-100 μmol m^{-2} s^{-1}, temperature 22 °C and relative humidity of 65%. When morning versus evening comparisons were performed (e.g., for PCD), two identical chambers with opposite-phased light regimens were used, so that the experiment was performed simultaneously on both sets of plants. For T1 transformants, the seedlings were grown on 1/2 Murashige and Skoog (MS) plates containing 0.8% agar, and 15 μg/mL Hygromycin B or 50 μg/mL Kanamycin for 10 days before being transplanted into soil and grown for 4 more weeks (5 weeks for *prr7 prr9*) before imaging. The transition to LL was performed either by changing the settings of the Percival chamber (along with the temperature adjustment) for the RASL-seq and glutathione measurements, by transferring the plants to a third Percival chamber (for PCD or qPCR experiments) or by placing plants in custom-built imaging chambers (for bioluminescence or fluorescence measurements).

Bacterial strains

Psm ES4326/*avrRpt2* was grown for two days on the King's Broth plates containing 1.5 % agar, 100 μg/mL of streptomycin, and 10 μg/mL tetracycline. *Agrobacterium tumefaciens* strain GV3101 was grown in LB media at 30 °C.

Plasmid construction

Cytoplasmic (Grx1-roGFP2), chloroplastic (TKTP-Grx1-roGFP2), and mitochondrial (SHMT-roGFP2-Grx1) roGFP2 constructs (10) were generously provided by Dr. A. Meyer. *CAT2p:LUC* and *CAT3p:LUC* constructs were described previously (11). Plasmid cloning for the luciferase reporter lines was performed using the Gateway Technology (Thermo Fisher Scientific). Specifically, the 818 bp and 1464 bp regions (including the 5' leader sequence) upstream of the start codons of *GSNOR1* and *G6PD6*, respectively, were amplified using the KOD DNA polymerase (EMD Millipore) and transferred into the pDONR207 plasmid using the BP recombination reaction and sequenced for accuracy. The resulting entry clone was then transferred to the destination vector pGWB535 (12) to obtain the final *GSNOR1p:LUC* and *G6PD6p:LUC* constructs using LR recombination. All cloning was performed using OneShot^{TM} TOP10 competent *E. coli* (Thermo Fisher Scientific). The final constructs were transformed into *Agrobacterium tumefaciens* strain GV3101 using the heat-shock method.

RNA extraction and RASL-seq

WT and *prr7 prr9* plants were transferred to LL at 22 °C or 30 °C, then, after 24 h, tissue was collected and frozen in liquid nitrogen every 4 h consecutively for 3 days and then pulverized using Geno/Grinder (Thermo Fisher Scientific). From the ground samples, RNA extraction was performed using the TRIZOL reagent (Ambion) according to manufacturer's instructions. The extraction was performed in batches of 18, with each batch containing a full 3-day time-course for a given genotype/condition. Probe annealing, ligation, and library construction was performed as described (2). All probe target sequences along with the respective gene identifiers can be found in Dataset S1. Illumina NovaSeq6000 sequencing was performed at Duke Center for Genomic and Computational Biology. The sequence alignment was performed using Bowtie2. Only probes with median of more than 10 reads (across all samples for a given genotype/temperature) were used for analysis. The reads were normalized to total counts and log-transformed to obtain normal distribution for statistical analysis. To account for occasional probe failure, for each timepoint a

possible outlier (defined as having a value outside two standard deviations from the median) was eliminated.

RT-qPCR

Reverse transcription (SuperScript IV, Invitrogen) and qPCR (SYBR Green, Roche) were performed according to the manufacturer's protocols. *UBIQUITIN 5* (*UBQ5*) mRNA was used for normalization. Primer sequences are provided in Table S1.

RASL-seq time-course data clustering and period estimation

A two-step approach was taken to identify possible redox rhythm-regulated genes. First, clustering of the genes based on their overall temporal signature was performed. The normalized mean traces for clustering were obtained with each batch of extraction (e.g., a set of 18 WT samples from 24 h to 92 h under LL) treated as a single replicate trace. The trace had its mean subtracted before being combined with the other replicates to eliminate the minor, but statistically significant batch effect for the average expression level (e.g., P = 0.02442 for *prr7 prr9* at 22 °C obtained by linear mixed model). To further ensure that the dominant signature of clustering was the oscillatory pattern, global trends were removed via cubically detrending the obtained mean and normalizing it to be between 0 and 1. The *k*-means clustering was performed on the means using MATLAB (MathWorks, version R2022a) while the hierarchical clustering was done using http://www.bioinformatics.com.cn/srplot, an online platform for data analysis and visualization. Second, the period distribution for genes within each cluster was obtained using either the CosinorPY package (v3.1) (13) or MetaCycle (14) (meta2d using combined JTK and LS methods with Fisher's test) with the periods set between 22 h and 36 h for all genotypes/conditions and only statistically significant periods ($p<0.05$) were selected.

For individual genes (*LHY*, *GSH1*, *FT*, *CAT2*, *GSNOR1*, *CAT3*, and *CAB2*) as well as total glutathione and GSSG, harmonic regression was performed using GraphPad Prism 9. The traces were decomposed into an exponentially decaying sine wave and a linear trendline (11):

$$X=A\times e^{-kt}\sin(2\pi\times(t+phase)/period)+b\times t+c,$$

where t is time in hours, A is the amplitude, and k is the decay rate. The standard error and degrees of freedom of period were used for statistical analysis.

Glutathione measurements

Leaf tissues for glutathione measurements were collected and handled concurrently with those for the RASL-seq experiment. The extraction and measurement were performed as described (15), with modifications. The samples were kept at or below 4 °C throughout the whole extraction process. Briefly, the ground tissue was homogenized using 1 mL ice-cold 0.2 M HCl per 100 mg tissue. The mixture was incubated on ice for 10 min, then spun down at 16000 g for 10 min using a refrigerated centrifuge. 200 mL of the supernatant was then transferred into new tubes, then 20 μL of 0.2 M NaH_2PO_4 (pH 5.6) was added, and the samples were adjusted to pH 5-6 using 0.2 M NaOH to measure the GSH+GSSG content. To measure GSSG, 150 μL aliquot of the total glutathione sample was incubated with 1% 2-vinylpyridine (Sigma) at room temperature for 45 min with occasional vigorous vortexing. Before the measurement, the samples were briefly centrifuged to remove the debris. Triplicate aliquots of the supernatant (10 μL for GSH+GSSG and 40 μL for GSSG) were transferred into a 96-well plate with each well containing 0.1 mL of 0.2 M NaH_2PO_4 with 10 mM EDTA (pH 7.5), 45 μL of water, 5 μL of 10 mM NADPH (Roche) and 10 μL of 12 mM 5,5′-dithiobis-(2-nitrobenzoic acid) (Sigma). Then, 0.2 U of freshly prepared glutathione reductase (GR) from *S. cerevisiae* (Sigma) was added and, after mixing, the increase in absorbance at 405 nm was monitored for at least 10 min using the Victor X5 plate reader (PerkinElmer). The slope of each sample was then normalized to one obtained from the standard curve using GSH (Sigma) to obtain the glutathione amount.

Mathematical modeling of redox rhythm phase shift in a long-period mutant

The 6-ODE mathematical model by del Olmo et al. (16) was modified by introducing a term for oscillating reduced glutathione being a scavenger of mitochondrial H_2O_2. Briefly, a term:

$$-g \times \cos\left(\frac{2\pi t}{T}\right),$$

where g is the coupling constant (equal to 0.06 for Fig. 2*A*) and T is the respective period (25.5 h for WT as the intrinsic period for the model and 36 h for the long period mutant), was added to the first equation of the 6-ODE model (16). The modeling was performed using Mathematica 13.3 (Wolfram Research, Inc., Champaign, IL, 2023).

Bioluminescence measurement and analysis

Plants of appropriate age were transferred from a Percival chamber to a custom-built imaging chamber and adapted for 48 h under LD during which they were sprayed with 2.5 mM luciferin (Gold Biotechnology) in 0.02% Triton X-100 (Sigma) at 24 h and 36 h after the transfer. Immediately before the start of the imaging, the light setting was switched from LD to LL. Images were taken every 2 h using a charge-coupled device camera (PIXIS 2048B) with the exposure time of 20 min. After the imaging, the means of the luminescence of shoot apex regions of the plants were calculated using the ImageJ software (17). The timepoints where the shoot apex was obscured by the emerging rosette leaves were excluded from the analysis. Each trace was then normalized to the maximum of its expression (the peak during the first day) to account for the intensity variability between different T1 transformants. The periods (set between 18 h and 38 h for all genotypes/reporters) were estimated using Biodare's FFT-NLLS algorithm (18) without detrending using the data from 24 h to 120 h under LL. All results with RAE $\leq$ 0.7 were considered rhythmic, combined with linear mixed-modeling, then compared through two-tailed Student's t-test.

Conductivity assay for measuring PCD

On the day of infection, single colonies were picked from plates and resuspended in 10 mM $MgSO_4$ to the final concentration of $OD_{600\ nm}$ = 0.02. To induce ETI-mediated PCD, youngest fully expanded leaves (three per plant) were pressure-infiltrated with *Psm* ES4326/*avrRpt2* 1.5 h after subjective dawn and at subjective dusk, for morning and evening infections, respectively. For dex-induced expression of AvrRpt2 to trigger PCD, solution of 0.25 μM of dex and 0.01 % Silwet L-77 (Plantmedia) with or without 30 μM GSHmee (Sigma) was sprayed to uniformly cover the foliage. Leaf discs (6 per sample) were collected using a 5 mm biopsy punch (Electron Microscopy

Sciences) 10 min after bacterial infiltration or 1 h after dex treatment and washed by floating in 30 mL deionized water for 45 min before being transferred into 6 mL of deionized water. Ion leakage was estimated by measuring conductivity using an Orion Star series meter (Thermo Scientific). Continuous ambient light was used throughout the measurement. After the last timepoint, the samples were boiled for 45 min to obtain the total conductivity for normalization.

Fluorescence imaging and analysis

The fluorescence chamber was custom-built from the Eppendorf CellXpert C170 Incubator with Cairn Incubator Control Unit and Cairn OPTOSpin Control Unit added. White LEDs provided constant low ambient light throughout the experiment, except when images were taken. The illumination for excitation was provided by the CoolLED pE-4000 system at 405 nm and 490 nm wavelengths for oxidized and reduced roGFP2 measurements, respectively. To avoid disrupting the clock, we used low light intensity (<3 μmol/m^{-2}, much lower than the intensity used for growth) for excitation, limited the exposure to 4 s and 10 s for 490 nm and 405 nm wavelengths, respectively, and took images every 10 min to minimize the total fluence. Fluorescence signals were collected at 535 nm with 30 nm bandwidth using the Andor iXon Ultra 888 EMCCD Camera. The images were then analyzed using ImageJ. The conversion of the fluorescence measurements to the degree of roGFP2 oxidation was previously done by a scaling factor determined through saturating tissue samples with H_2O_2 or DTT (19), a method that is not applicable on mature plants. Therefore, the fluorescence ratio of signals from excitations at 490 nm over 405 nm was normalized to the initial timepoint to track the change in dynamics (10). For the redox rhythm measurement, the periods (set between 18 h and 38 h for both WT and *prr7 prr9*) were estimated using Biodare's FFT-NLLS algorithm (18) without detrending using the data from 48 h to 120 h under LL. All results with RAE $\leq$ 0.7 were considered rhythmic.

For microscopy, the infection process was monitored continuously in the fluorescence imaging chamber with images taken every 10 min, and leaf discs were collected at relevant infection stages for confocal laser scanning microscopy. The imaging was done with the Zeiss 880 airyscan inverted microscope using a 20× objective lens. Images were collected in multi-track mode

with line switching between 405 nm and 488 nm excitations. The roGFP2 fluorescence was collected between a band pass filter of 498–525 nm.

The images were analyzed using ImageJ. Cytoplasmic and chloroplastic roGFP2 images were segmented manually, while for mitochondrial roGFP2, fluorescent puncta were identified using the find maxima function with the prominence value set to >50.

Statistical analysis and data processing

All statistical analyses and harmonic regression were done using GraphPad Prism 9. Data processing was done in MATLAB (MathWorks, version R2022a), while for linear mixed-effect modeling, lmer package was used in R (version 4.1.2). Gene ontology enrichment analysis was performed using ShinyGO version 0.77 (20) (http://bioinformatics.sdstate.edu/go/) and the GO terms with false discovery rate (FDR) value less than 0.05 were selected. Redundant GO terms, defined as having more than 90 % overlap with the most significant GO term, were removed before plotting. In the graphs, asterisks indicate statistical significance reflecting the *P* values (* $P < 0.05$, ** $P < 0.01$, *** $P < 0.001$, **** $P < 0.0001$, and ns, not significant). Unless specified, experiments were repeated at least three times with similar results.

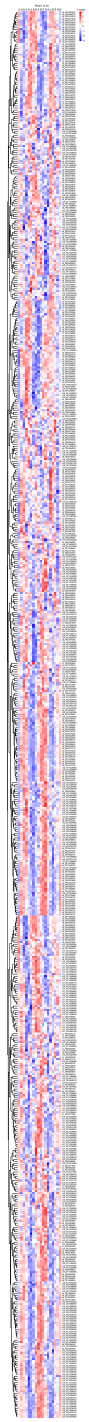

Fig. S1. Deterministic clustering supports the results from *k*-means clustering. The heatmap shows the hierarchical clustering of the RASL-seq dataset from the *prr7 prr9* at 22 °C samples based on Euclidean distance using complete linkage. Zoom to see individual transcripts. The columns represent the timepoints while the rows are labeled with the gene model identifier preceded with the cluster number assigned by the *k*-means clustering analysis.

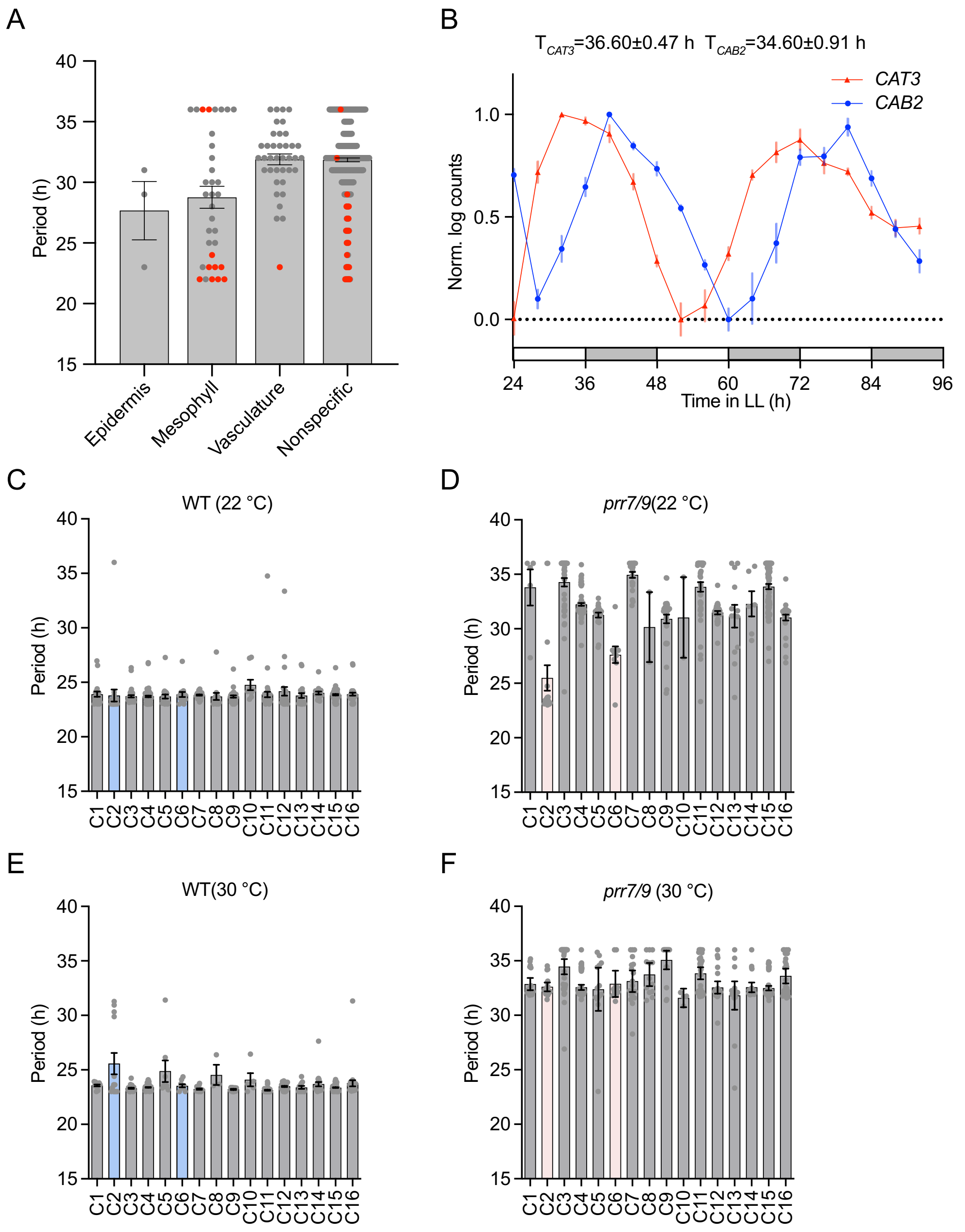

Fig. S2. Period separation in *prr7 prr9* is not due to tissue-specific effects nor algorithm-specific. (*A*) Period estimates using CosinorPY of the genes from the RASL-seq data with and without tissue-specificity according to Berkowitz et al. (21). n = 3, 34, 38 and 464 genes for epidermis, mesophyll, vasculature, and nonspecific genes, respectively. Periods for C2 and C6 genes are labeled in red. (*B*) Normalized expression of *CAT3* and *CAB2* in *prr7 prr9* at 22 °C. n ≥ 5 biological

replicates per timepoint. The periods were determined using harmonic regression. (*C*-*F*) Periods of oscillatory transcripts in WT (*C* and *E*) and *prr7 prr9* (*prr7/9*) (*D* and *F*) at 22 °C (*C* and *D*) and 30 °C (*E* and *F*). Clusters were generated based on *k*-means analysis of expression signatures of all transcripts in the RASL-seq dataset from the *prr7/9* at 22 °C samples. For each cluster, periods of statistically significant ($p<0.05$) oscillatory transcripts in WT and *prr7 prr9* were estimated using MetaCycle, with each dot representing the period of a single transcript. Clusters of interest, C2 and C6, are colored blue (*C* and *E*) and red (*D* and *F*), respectively. All values are means ± SEM.

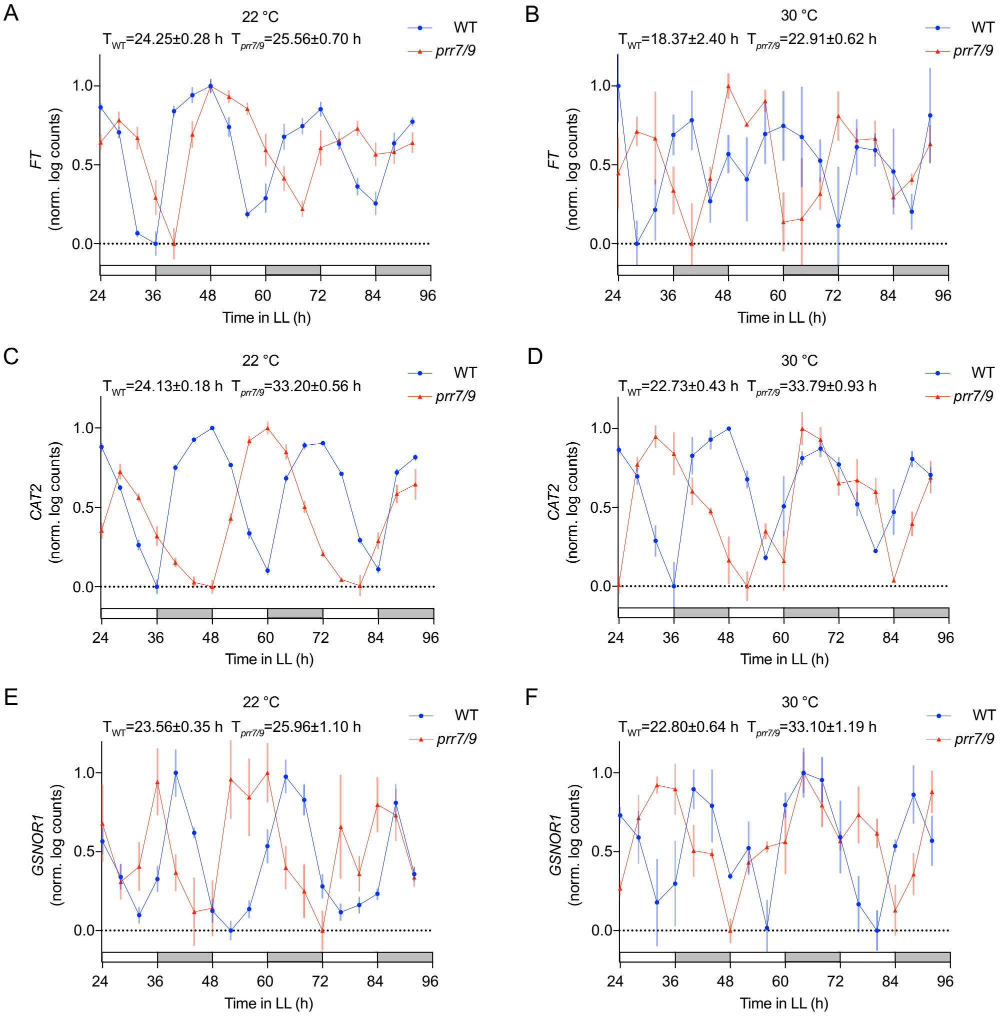

Fig. S3. Higher temperature delinks *GSNOR1,* but not *FT*, from the redox rhythm. (*A-F*) Normalized expressions of *FT* (*A* and *B*), *CAT2* (*C* and *D*), and *GSNOR1* (*E* and *F*) from RASL-seq at 22 °C (left) and 30 °C (right) with estimated oscillation periods (T_{WT} and $T_{prr7/9}$). n =5 or 6 biological replicates per timepoint for 22 °C and n = 3 biological replicates per timepoint for 30 °C. The periods were determined using harmonic regression. All values are means ± SEM.

A

Cluster 2	Cluster 3	Cluster 4
Response to biotic stimulus	Photosynthesis	Peptide biosynthetic proc.
Programmed cell death	Photosynthesis, light reaction	Organonitrogen compound biosynthetic proc.
Abscission	Reg. of photosynthesis	Ribonucleoprotein complex biogenesis
Pos. reg. of defense response	Reg. of photosynthesis, light reaction	Ribosome assembly
Defense response	Photosystem II stabilization	Ribonucleoprotein complex assembly

B

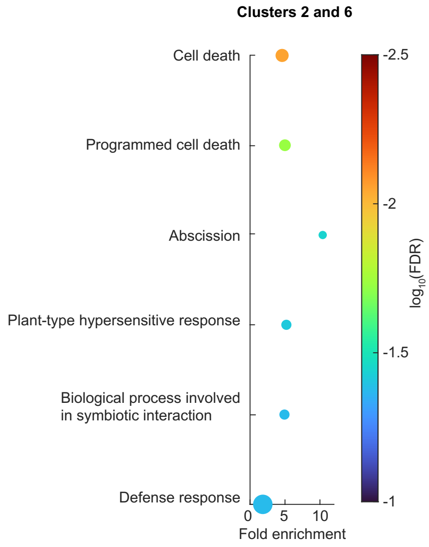

C

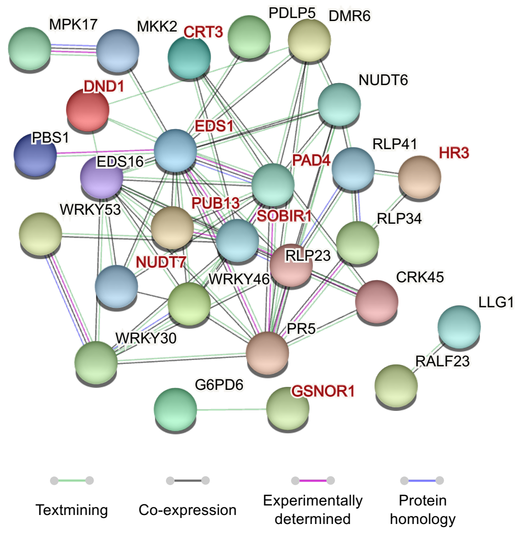

Fig. S4. The analysis of only genes with statistically significant oscillations supports the regulation of PCD by the redox rhythm. (*A*) Top 5 GO terms based on the false discovery rate (FDR) for all oscillatory ($p<0.05$) genes in individual clusters with significant enrichment using the total RASL-seq gene pool (~700 genes) as the background. (*B*) GO terms for combined oscillatory C2 and C6 genes, sorted by FDR. (*C*) StringDB analysis for oscillatory C2 and C6 genes using textmining, co-expression, experimental evidence, and protein homology. Dark red indicates cell death genes. Interaction score was set to 0.400.

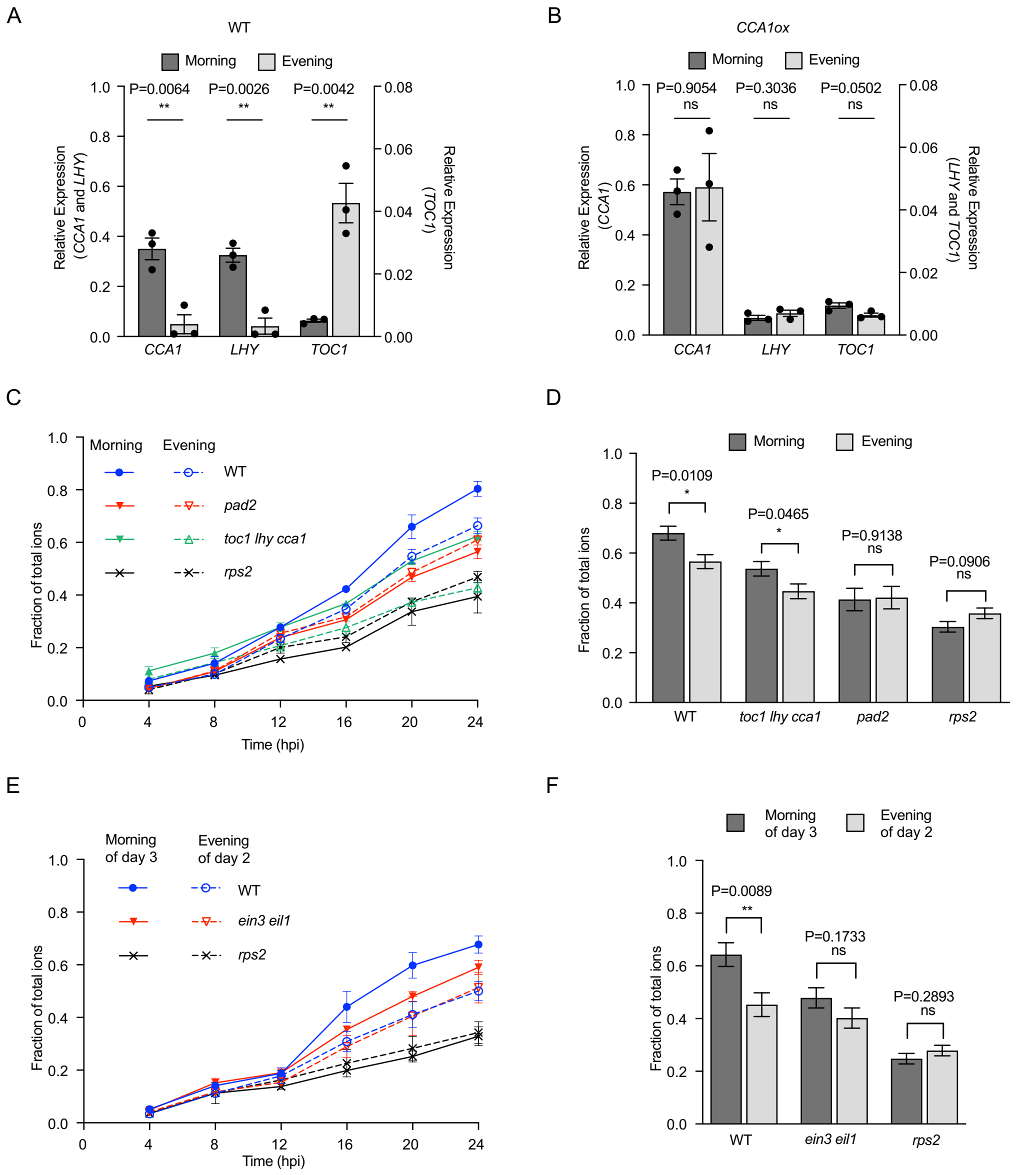

Fig. S5. Redox rhythm, but not genetic clock or light exposure duration, drives the time-of-day sensitivity of ETI-induced PCD. (*A* and *B*) The relative expression of core clock genes *CCA1*, *LHY*, and *TOC1* mRNA in WT (*A*) and *CCA1ox* (*B*) after 2 days under LL in the subjective morning and the subjective evening. Transcript abundance was measured using qPCR and the clock gene levels were normalized to the expression of *UBIQUITIN5*. The values were compared using two-tailed Student's t-test; ns, not significant. n = 3 biological replicates. The experiment has been repeated 3 times with similar results. (*C* and *D*) Time-course of ion leakage (a measure of cell death). After infiltration with *Psm* ES4326/*avrRpt2* in WT, *pad2*, *toc1 lhy cca1*, and *rps2* ion leakage was

estimated by measuring conductivity at each timepoint normalized to conductivity of total ions for each sample (*C*). Analysis of normalized conductivity at 20 hours post infiltration (hpi) combined from three separate experiments using linear mixed-effect model (*D*). Two-tailed Student's t-test, ns, not significant. (*E* and *F*) Time-course of ion leakage measured as in (*C*) in WT, *ein3 eil1*, and *rps2* on the morning of the third day and the evening of the second day under LL (*E*). Normalized conductivity at 20 hpi from three separate experiments (*F*) was analyzed as in (*D*). n = 3 biological replicates per timepoint for (*C* and *E*), and n = 9 biological replicates pooled from three separate experiments for (*D* and *F*). All values are means ± SEM.

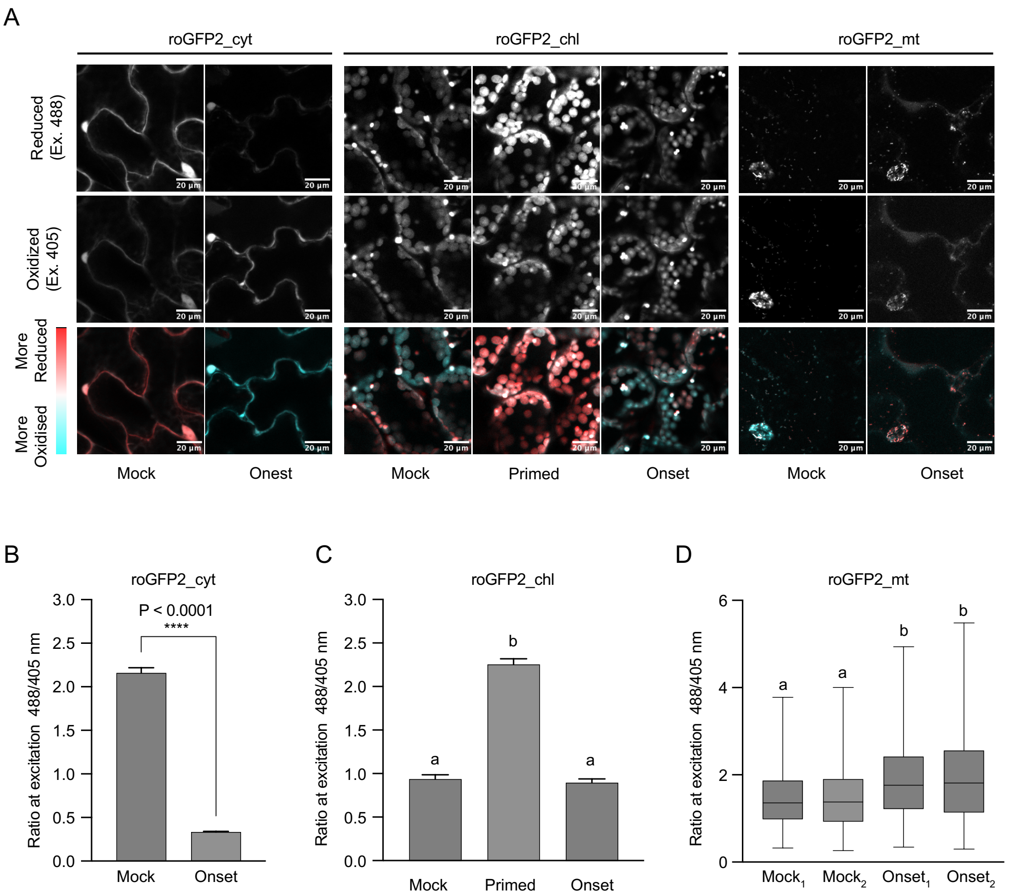

Fig. S6. Subcellular compartments show differing redox potential leading to ETI-mediated PCD. (*A*) Representative images of confocal micrographs for mock or *Psm* ES4326/*avrRpt2*- infiltrated leaves chosen from the "primed" or "PCD" stage during whole-plant time-course fluorescence imaging as in Fig. 5 *E* and *F*. The "primed" stage, the peak time of reduced roGFP2_chl signal. The "PCD" stage, the time immediately before tissue collapse. Top row, reduced roGFP2 (excitation at 488 nm); middle row, oxidized roGFP2 (excitation at 405 nm); and bottom row, false color combination of the images with reduced and oxidized roGFP2 images represented by red and cyan colors, respectively. (*B*) Quantification of roGFP2_cyt from (*A*). The values were compared using two-tailed Student's t-test. n = 60 different positions. (*C*) Quantification of roGFP2_chl from (*A*). The values were compared using ANOVA with Tukey's post hoc, P = 0.8538 comparing a to a, and P<0.0001 comparing a to b. n = 59 chloroplasts from each condition. (*D*) Quantification of all puncta from 4 different micrographs for roGFP2_mt. The values were compared using ANOVA with Tukey's post hoc, P = 0.9895 comparing a to a, P = 0.6541 comparing b to b, and P<0.0001

comparing a to b. n = 2322, for $Mock_1$, n = 2686 for $Mock_2$, n = 1768 for PCD_1 and n = 2017 for PCD_2 puncta per image. The experiment has been repeated 3 times with similar results. Values are means ± SEM for (*B* and *C*) and medians with interquartile range with whiskers representing full range for (*D*).

Table S1. Primers used in this study.

Primer Name	Sequence (5'->3')	Purpose	Source
GSNOR1p_F	AAAAAGCAGGCTCGTGCTAAACCTCAGCAAAATCATGTG	Cloning	This study
GSNOR1p_R	AGAAAGCTGGGTGTGACGCAGGAAGAAATGAGAG	Cloning	This study
G6PD6p_F	AAAAAGCAGGCTCGTGCCTCCCCGTCCTGAC	Cloning	This study
G6PD6p_R	AGAAAGCTGGGTGCTCCTTAAAACCTTAATTTCCACAC	Cloning	This study
attB1ad_F	GGGGACAAGTTTGTACAAAAAAGCAGGCT	Cloning	Universal primer
attB2ad_R	GGGGACCACTTTGTACAAGAAAGCTGGGT	Cloning	Universal primer
pDONR207F	TCGCGTTAACGCTAGCATGGATCTC	Sequencing	Universal primer
pDONR207R	GTAACATCAGAGATTTTGAGACAC	Sequencing	Universal primer
UBQ5_qP_F	GACGCTTCATCTCGTCC	qPCR	Zhou, Wang et al., 2015
UBQ5_qP_R	GTAAACGTAGGTGAGTCCA	qPCR	Zhou, Wang et al., 2015
TOC1_qP_F	AATAGTAATCCAGCGCAATTTTCTTC	qPCR	Zhou, Wang et al., 2015
TOC1_qP_R	CTTCAATCTACTTTTCTTCGGTGCT	qPCR	Zhou, Wang et al., 2015
LHY_qP_F	CGCTGCTTCGGTCTGGCCTT	qPCR	Zhou, Wang et al., 2015
LHY_qP_R	TGTAGCAGCGGCAATGGCAGT	qPCR	Zhou, Wang et al., 2015
CCA1_qP_F	TGACCGGTCCTCGTGTGGCT	qPCR	Zhou, Wang et al., 2015
CCA1_qP_R	ACTGCGGCGTGCATTGGACT	qPCR	Zhou, Wang et al., 2015

Citations

1. M. Mwimba *et al.*, Daily humidity oscillation regulates the circadian clock to influence plant physiology. *Nature Communications* **9**, 4290 (2018).
2. W. Wang *et al.*, Timing of plant immune responses by a central circadian regulator. *Nature* **470**, 110-U126 (2011).
3. R. Zavaliev, R. Mohan, T. Chen, X. Dong, Formation of NPR1 Condensates Promotes Cell Survival during the Plant Immune Response. *Cell* **182**, 1093-1108.e1018 (2020).
4. L. Liu *et al.*, Salicylic acid receptors activate jasmonic acid signalling through a non-canonical pathway to promote effector-triggered immunity. *Nat Commun* **7**, 13099 (2016).
5. J. Glazebrook, F. M. Ausubel, Isolation of phytoalexin-deficient mutants of Arabidopsis thaliana and characterization of their interactions with bacterial pathogens. *Proceedings of the National Academy of Sciences* **91**, 8955-8959 (1994).
6. Y. Xiong, C. DeFraia, D. Williams, X. Zhang, Z. Mou, Characterization of Arabidopsis 6-Phosphogluconolactonase T-DNA Insertion Mutants Reveals an Essential Role for the Oxidative Section of the Plastidic Pentose Phosphate Pathway in Plant Growth and Development. *Plant and Cell Physiology* **50**, 1277-1291 (2009).
7. J. M. Alonso *et al.*, Five components of the ethylene-response pathway identified in a screen for weak ethylene-insensitive mutants in Arabidopsis. *Proc Natl Acad Sci U S A* **100**, 2992-2997 (2003).
8. O. Lorenzo, J. M. Chico, J. J. Sánchez-Serrano, R. Solano, JASMONATE-INSENSITIVE1 encodes a MYC transcription factor essential to discriminate between different jasmonate-regulated defense responses in Arabidopsis. *Plant Cell* **16**, 1938-1950 (2004).
9. S. J. Harrison *et al.*, A rapid and robust method of identifying transformed Arabidopsis thaliana seedlings following floral dip transformation. *Plant Methods* **2**, 19 (2006).
10. J. M. Ugalde *et al.*, Chloroplast-derived photo-oxidative stress causes changes in H2O2 and EGSH in other subcellular compartments. *Plant Physiology* **186**, 125-141 (2021).
11. M. Zhou *et al.*, Redox rhythm reinforces the circadian clock to gate immune response. *Nature* **523**, 472-476 (2015).
12. T. Nakagawa *et al.*, Improved Gateway binary vectors: high-performance vectors for creation of fusion constructs in transgenic analysis of plants. *Biosci Biotechnol Biochem* **71**, 2095-2100 (2007).
13. M. Moškon, CosinorPy: a python package for cosinor-based rhythmometry. *BMC Bioinformatics* **21**, 485 (2020).
14. G. Wu, R. C. Anafi, M. E. Hughes, K. Kornacker, J. B. Hogenesch, MetaCycle: an integrated R package to evaluate periodicity in large scale data. *Bioinformatics* **32**, 3351-3353 (2016).
15. G. Queval, G. Noctor, A plate reader method for the measurement of NAD, NADP, glutathione, and ascorbate in tissue extracts: Application to redox

profiling during Arabidopsis rosette development. *Analytical Biochemistry* **363**, 58-69 (2007).
16. M. del Olmo, A. Kramer, H. Herzel, A Robust Model for Circadian Redox Oscillations. *Int J Mol Sci* **20** (2019).
17. C. A. Schneider, W. S. Rasband, K. W. Eliceiri, NIH Image to ImageJ: 25 years of image analysis. *Nature Methods* **9**, 671-675 (2012).
18. T. Zielinski, A. M. Moore, E. Troup, K. J. Halliday, A. J. Millar, Strengths and Limitations of Period Estimation Methods for Circadian Data. *PLOS ONE* **9**, e96462 (2014).
19. A. J. Meyer *et al.*, Redox-sensitive GFP in Arabidopsis thaliana is a quantitative biosensor for the redox potential of the cellular glutathione redox buffer. *Plant J* **52**, 973-986 (2007).
20. S. X. Ge, D. Jung, R. Yao, ShinyGO: a graphical gene-set enrichment tool for animals and plants. *Bioinformatics* **36**, 2628-2629 (2020).
21. O. Berkowitz *et al.*, RNA-seq analysis of laser microdissected Arabidopsis thaliana leaf epidermis, mesophyll and vasculature defines tissue-specific transcriptional responses to multiple stress treatments. *The Plant Journal* **107**, 938-955 (2021).
